# A leader supply-demand-based optimization for large scale optimal power flow problem considering renewable energy generations

**DOI:** 10.1038/s41598-023-41608-1

**Published:** 2023-09-04

**Authors:** Fatima Daqaq, Mohamed H. Hassan, Salah Kamel, Abdelazim G. Hussien

**Affiliations:** 1 Laboratory of Study and Research for Applied Mathematics, Mohammadia School of Engineers, Mohammed V University in Rabat, Rabat, 10090 Morocco; 2 Ministry of Electricity and Renewable Energy, Cairo, Egypt; 3 Department of Electrical Engineering, Faculty of Engineering, Aswan University, Aswan, 81542 Egypt; 4 Department of Computer and Information Science, Linköping University, Linköping, Sweden; 5 Faculty of Science, Fayoum University, Faiyum, Egypt; 6 MEU Research Unit, Faculty of Information Technology, Middle East University, Amman, Jordan

**Keywords:** Engineering, Electrical and electronic engineering, Mathematics and computing, Computer science

## Abstract

The supply-demand-based optimization (SDO) is among the recent stochastic approaches that have proven its capability in solving challenging engineering tasks. Owing to the non-linearity and complexity of the real-world IEEE optimal power flow (OPF) in modern power system issues and like the existing algorithms, the SDO optimizer necessitates some enhancement to satisfy the required OPF characteristics integrating hybrid wind and solar powers. Thus, a SDO variant namely leader supply-demand-based optimization (LSDO) is proposed in this research. The LSDO is suggested to improve the exploration based on the simultaneous crossover and mutation mechanisms and thereby reduce the probability of trapping in local optima. The LSDO effectiveness has been first tested on 23 benchmark functions and has been assessed through a comparison with well-regarded state-of-the-art competitors. Afterward, Three well-known constrained IEEE 30, 57, and 118-bus test systems incorporating both wind and solar power sources were investigated in order to authenticate the performance of the LSDO considering a constraint handling technique called superiority of feasible solutions (SF). The statistical outcomes reveal that the LSDO offers promising competitive results not only for its first version but also for the other competitors.

## Introduction

During the past decades, optimization has aroused an increase due to its importance in various fields including engineering design, economics, computer science, business, operational research, etc. Besides, the most popular real word optimization problem is the optimal power flow in power system operation and planning^1^. The OPF is regarded as a high-dimensional, non-convex, non-linear, complex issue. Solving the OPF problem efficiently and accurately plays a vital role in power system operation and planning. By achieving an optimal dispatch of generation resources, OPF helps to improve system efficiency. Additionally, OPF enables the integration of renewable energy sources, enhances grid resilience, and facilitates the reliable and secure operation of power systems, thereby ensuring the provision of reliable and affordable electricity to consumers. Furthermore, the primary objective function is minimizing fuel cost, then the emission, voltage deviation, power loss, etc, taking into account numerous constraints on generators, bus voltage, line capacity, transformer tap, and also active and reactive power of generators, which should be satisfied. Moreover, the OPF problem can be mainly solved via two categories of optimization techniques: the first one is classical or deterministic approaches that converged to local optima and suffered from convexity. The second is the intelligent or stochastic approaches that are considered an effective methods for finding optimal solutions. In general, many scholars have been successfully applied various stochastic approaches to address the power system issues including adaptive constraint differential evolution (ACDE) algorithm^2^, an improved version of the coyote optimization algorithm (COA)^3^, teaching-learning-based optimizer (TLBO)^4^, adaptive multiple teams perturbation-guiding Jaya (AMTPG-Jaya)^5^, backtracking search algorithm (BSA)^6^, crisscross search based grey wolf optimizer (CS-GWO)^7^, ant colony optimization (ACO)^8^, effective whale optimization algorithm (EWOA)^9^, moth swarm algorithm (MSA)^10^, adaptive group search optimization (AGSO)^11^, improved colliding bodies optimization (ICBO)^12^, differential search algorithm (DSA)^13^, invasive weed optimization (IWO)^14^, interior search algorithm (ISA)^15^, robust optimization approach (Rao)^16^, Salp swarm algorithm (SSA)^17^. Stud krill herd algorithm (SKH)^18^, symbiotic organisms search algorithm (SOS)^19^, tree-seed algorithm (TSA)^20^, Hunter-prey optimization (HPO)^21^, particle swarm optimization (PSO)^22^, fuzzy-based improved comprehensive-learning particle swarm optimization (FBICLPSO) algorithm^23^, hybrid Grey wolf optimizer and particle swarm optimization (GWO-PSO)^24^, hybrid of the firefly and PSO algorithms (HFAPSO)^25^, combined genetic algorithm and particle swarm algorithm (GA-PSO)^26^, multi objective genetic algorithm (MOGA)^27^, artificial bee colony algorithm based on a non-dominated sorting genetic approach (ABC-NSGA-II)^28^, fitness-distance balance based-TLABC (teaching-learning-based artificial bee colony) (FDB-TLABC)^29^, non-dominated sorting culture differential evolution algorithm (NSCDE)^30^, differential evolution algorithm based on state transition of specific individuals (DE-TSA)^31^, multi-objective covariance matrix adaptation evolution strategy (CMA-ES)^32^, manta ray foraging optimization (MRFO)^33,34^, dragonfly algorithm (DA)^35^, flower pollination algorithm (FPA)^36^, etc.

Therefore, the aim of this current work is to improve the SDO algorithm in order to apply it to the OPF IEEE 30-bus, IEEE 57-bus, and IEEE 118-bus test power systems with and without considering hybrid Wind/Solar energy resources. Besides, the implementation of the SDO optimizer to deal with OPF issues is investigated for the first time. The SDO approach is a novel stochastic optimizer, introduced by Zhao et al. in 2019 and inspired by the supply-demand mechanism in economics^37^. Numerous academic researchers have employed the SDO algorithm such as, in Refs.^38,39^ the authors apply SDO in order to extract accurate and reliable parameters for different PV models. To design an efficient and economic hybrid energy system, the SDO optimizer was used in^40^. According to^41^, the fitness-distance balance (FDB) method was employed to effectively model the supply-demand processes in SDO. Additionally, in order to build an accurate equivalent circuit model for proton exchange membrane fuel cells, authors in^42^ tried to apply the SDO algorithm. As introduced in^43^, the authors apply the SDO in order to obtain the unknown parameters of the PIDA controller. Referring to^44^, Hassan et all. improve SDO with a view to enhance the population diversity, the balance between local and global search, and the premature convergence of the original supply-demand based optimization (SDO) algorithm. Their proposed approach was applied for achieving global solutions to economic load dispatch (ELD) problems in power systems. In addition, in an attempt to ameliorate the performance of the approach under study, the authors in^45^ present a chaotic map-based supply-demand optimization (SDO) algorithm including the fitness-distance balance (FDB) selection method to solve the Combined heat and power economic dispatch (CHPED) problem; the FDB and chaotic maps were used to increase the convergence performance of the algorithm to the global solution and to find the global solution in the solution search space. Regarding the work of Zhao et al.^46^, an enhanced fitness-distance balance (EFDB) and the Levy flight are added to the SDO original version to avoid premature convergence and improve solution diversity; besides, a mutation mechanism is introduced into the algorithm to improve search efficiency; and to enhance the convergence accuracy, an adaptive local search strategy (ALS) is integrated, and so on. According to these literature reviews, the supply-demand-based optimization algorithm requires an adjustment in terms of the exploration behavior to fit the current problem. This has motivated us to suggest the leader supply-demand-based optimization approach (LSDO). Thus, during each SDO’ generation a leader-based mutation selection adaptively perched over the exploration phase.

The contributions of this paper are:

- The proposed LSDO algorithm is evaluated by testing it on various benchmark functions. It is compared against established algorithms such as Social Network Search (SNS), Gray Wolf Optimizer (GWO), Tunicate Swarm Algorithm (TSA), and the original SDO algorithm. This evaluation helps assess the performance and effectiveness of the LSDO algorithm.
- The LSDO algorithm is implemented to solve the Optimal Power Flow (OPF) problem on three well-known standard systems: IEEE 30-bus, IEEE 57-bus, and IEEE 118-bus test systems considering Wind and Solar powers. These systems have different numbers of control variables (24, 33, and 130, respectively). By considering these standard systems, the paper ensures a comprehensive evaluation of the LSDO algorithm’s capabilities.
- Comparative studies are conducted between the proposed LSDO technique and the original SDO technique for solving the OPF problem. By comparing these two approaches, the paper aims to highlight the advantages and improvements achieved by the LSDO algorithm.
- The OPF problem is solved using both the proposed LSDO and the original SDO techniques in eight different cases with single objectives. These objectives include total cost minimization, total emission minimization, active power loss minimization, and voltage deviation minimization. By addressing these different objectives, the paper demonstrates the versatility and applicability of the LSDO algorithm in tackling various aspects of the OPF problem.
- Through comparative analysis, the paper shows that the proposed LSDO technique exhibits high robustness and outperforms the conventional SDO algorithm and other recent techniques in addressing the OPF problem. This analysis highlights the superior performance of the LSDO algorithm and its potential as a powerful optimization tool.

Overall, the paper contributes to the field by evaluating the performance of the LSDO algorithm, demonstrating its effectiveness in solving the OPF problem incorporating wind/solar powers, and showcasing its robustness and improved performance compared to existing techniques.

The following sections of this paper are organized as follows: In The proposed optimization methodology section, you will find a detailed explanation of the original SDO, and its improved variant LSDO, besides a brief introduction of the constraint handling strategy SF. Problem Formulation Methodology section introduces the formulation of the OPF problem considering renewable energy resources. Simulation Results and Discussion section of this paper delves into a comprehensive numerical statistical analysis and discussions. Ultimately, the paper concludes with a summary of the findings.

## The proposed optimization methodology

In this section, the supply-demand-based optimization (SDO) algorithm is briefly explained then the process of the leader SDO (LSDO) algorithm is described.

### The supply-demand-based optimization (SDO) algorithm

According to the SDO algorithm proposed in^37^, it is presumed that there exist multiple markets for commodities, each with a consistent quantity and cost for every product. The cost of each commodity and the corresponding market volume is presented as follows:

1 $$\begin{aligned} X= & {} \left[ \begin{array}{c} x_{1} \\ x_{2} \\ \cdot \\ \cdot \\ \cdot \\ x_{n} \end{array}\right] =\left[ \begin{array}{ccccc} x_{1}^{1} &{}\quad x_{1}^{2} &{}\quad \cdots &{}\quad \cdots &{}\quad x_{1}^{d} \\ x_{2}^{1} &{}\quad x_{2}^{2} &{}\quad \cdots &{}\quad \cdots &{} x_{2}^{d} \\ \vdots &{}\quad \vdots &{}\quad \vdots &{}\quad \vdots &{}\quad \vdots \\ \vdots &{}\quad \vdots &{}\quad \vdots &{}\quad \vdots &{}\quad \vdots \\ x_{n}^{1} &{}\quad x_{n}^{2} &{}\quad \cdots &{}\quad \cdots &{}\quad x_{n}^{d} \end{array}\right] \end{aligned}$$

2 $$\begin{aligned} Y= & {} \left[ \begin{array}{c} y_{1} \\ y_{2} \\ \cdot \\ \cdot \\ \cdot \\ y_{n} \end{array}\right] =\left[ \begin{array}{ccccc} y_{1}^{1} &{}\quad y_{1}^{2} &{}\quad \cdots &{}\quad \cdots &{}\quad y_{1}^{d} \\ y_{2}^{1} &{}\quad y_{2}^{2} &{}\quad \cdots &{}\quad \cdots &{}\quad y_{2}^{d} \\ \vdots &{}\quad \vdots &{}\quad \vdots &{}\quad \vdots &{}\quad \vdots \\ \vdots &{}\quad \vdots &{}\quad \vdots &{}\quad \vdots &{}\quad \vdots \\ y_{n}^{1} &{}\quad y_{n}^{2} &{}\quad \cdots &{}\quad \cdots &{}\quad y_{n}^{d} \end{array}\right] \end{aligned}$$

where *d* refers to the commodity prices number while *n* denotes the markets number. Moreover, $x_{i}^{j}(i=1,\ldots ,n; j=1,\ldots ,d)$ represents the *j*th commodity cost in the $i{\text {th}}$ market and $x_i(i=1,\ldots ,n)$ refers to the $i{\text {th}}$ the vector of commodity cost. $y_{i}^{j}(i=1,\ldots ,n; j=1,\ldots ,d)$ represents the *j*th commodity quantity in the *i*th market. $y_{i}(i=1,\ldots ,n)$ denotes the *i*th the vector of the commodity quantity.

The values of the decision variable in the fitness function are determined by the cost and quantity of commodities for each market, which are evaluated as follows:

3 $$\begin{aligned} \left[ \begin{array}{l} F_{x} \\ F_{y} \end{array}\right] =\left[ \begin{array}{llll} F_{x 1} &{}\quad F_{x 2} &{}\quad \ldots &{} F_{x n} \\ F_{y 1} &{}\quad F_{y 2} &{}\quad \ldots &{} F_{y n} \end{array}\right] ^{T} \end{aligned}$$

where *T* denotes the transpose of the matrix.

To prevent the SDO algorithm from becoming trapped in local optima, the balance costs $y_{0}$ and balance volume vector $x_{0}$ are chosen randomly, with a probability distribution determined by their likelihood of being successful.

4 $$\begin{aligned} N_{i}= & {} \left\| F_{y i}-\frac{1}{n} \sum _{i=1}^{n} F_{y i}\right\| \end{aligned}$$

5 $$\begin{aligned} Q= & {} \frac{N_{i}}{\sum \limits _{i=1}^{n} N_{i}} \end{aligned}$$

6 $$\begin{aligned} M_{i}= & {} \left\| F_{x i}-\frac{1}{n} \sum _{i=1}^{n} F_{x i}\right\| \end{aligned}$$

7 $$\begin{aligned} P= & {} \frac{M_{i}}{\sum \limits _{i=1}^{n} M_{i}} \end{aligned}$$

8 $$\begin{aligned} x_{o}= & {} {\left\{ \begin{array}{ll}r_{1} \sum _{1}^{n} \frac{x_{i}}{n} &{} \text{ if } \text{ rand } <0.5 \\ x_{k}, \,\,k= \text{ Roulette } \text{ wheel } \text{ selection } (FP) &{} \text{ if } \text{ rand } \ge 0.5\end{array}\right. } \end{aligned}$$

The quantities and costs of the product presented below are adjusted using the supply-factor $\alpha$ and demand-factor $\beta$, which are determined based on the equilibrium cost and balance quantity:

9 $$\begin{aligned} y_{i}(t+1)= & {} y_{o}+\alpha \cdot \left( x_{i}(t)-x_{0}\right) \end{aligned}$$

10 $$\begin{aligned} x_{i}(t+1)= & {} x_{o}+\beta \cdot \left( y_{i}(t)-y_{0}\right) \end{aligned}$$

During the *i*th iteration, $x_{i}(t)$ and $y_{i}(t)$ represent the *i*th cost and total quantity of a given product. The cost of the commodity can be expressed as:

11 $$\begin{aligned} x_{i}(t+1)=x_{o}+ L \cdot \left( x_{i}(t)-x_{0}\right) \end{aligned}$$

In order to balance exploration and exploitation, alpha and beta are denoted as:

12 $$\begin{aligned} \alpha= & {} \frac{2(T-t+1)}{T} \sin (2 \pi r) \end{aligned}$$

13 $$\begin{aligned} \beta= & {} 2 \cos (2 \pi r) \end{aligned}$$

here *t* refers to the current iteration, *r* is a random vector, and *T* denotes the total number of iterations.

To facilitate an efficient transition between exploration and exploitation within the SDO technique, a novel variable *L* is formulated as follows:

14 $$\begin{aligned} L=\alpha \beta =\frac{2(T-t+1)}{T} \sin (2 \pi r) \cos (2 \pi r) \end{aligned}$$

The cost of each demand varies between the balance cost when $|L|>1$, and the converged balance cost when $|L|<1$.

### The proposed leader supply-demand-based optimization (LSDO) algorithm

The proposed technique is called Leader-based mutation-selection^47^. Its purpose is to address the possibility of the optimal value falling into local optima. This approach involves using the best location vector $x_{best}^{t}$, the second-best location vector $x_{(best-1)}^{t}$, and the third-best location vector $x_{(best-2)}^{t}$ based on the objective function value of the new location vector $x_{i}(new)$ relative to the population size. The new mutation position vector $x_{i}(mut)$ is then calculated as:

15 $$\begin{aligned} \begin{aligned} x_{i}(mut)=&x_{i}(new)+2 \left( 1-\frac{t}{Max_it}\right) (2 rand -1)\left( 2 x_{best}^{t}-\left( x_{best-1}^{t}+x_{best-2}^{t}\right) \right) \\&+(2 \times rand -1)\left( x_{best}^{t}-x_{i}(new)\right) \end{aligned} \end{aligned}$$

Then, the next location is updated using the following equation^48^:

16 $$\begin{aligned} x_{i}(t+1)= {\left\{ \begin{array}{ll}x_{i}( \text{ mut } ) &{} f\left( x_{i}( \text{ mut } )\right) <f\left( x_{i}( \text{ new } )\right) \\ x_{i}( \text{ new } ) &{} f\left( x_{i}( \text{ mut } )\right) \ge f\left( x_{i}( \text{ new } )\right) \end{array}\right. } \end{aligned}$$

Finally, the optimal solution can be updated as follows^49^:

17 $$\begin{aligned} x_{\text{ best } }= {\left\{ \begin{array}{ll}x_{i}( \text{ mut } ) &{} f\left( x_{i}( \text{ mut } )\right)<f\left( x_{\text{ best } }\right) \\ x_{i}( \text{ new } ) &{} f\left( x_{i}( \text{ new } )\right) <f\left( x_{\text{ best } }\right) \end{array}\right. } \end{aligned}$$

**Figure 1:**
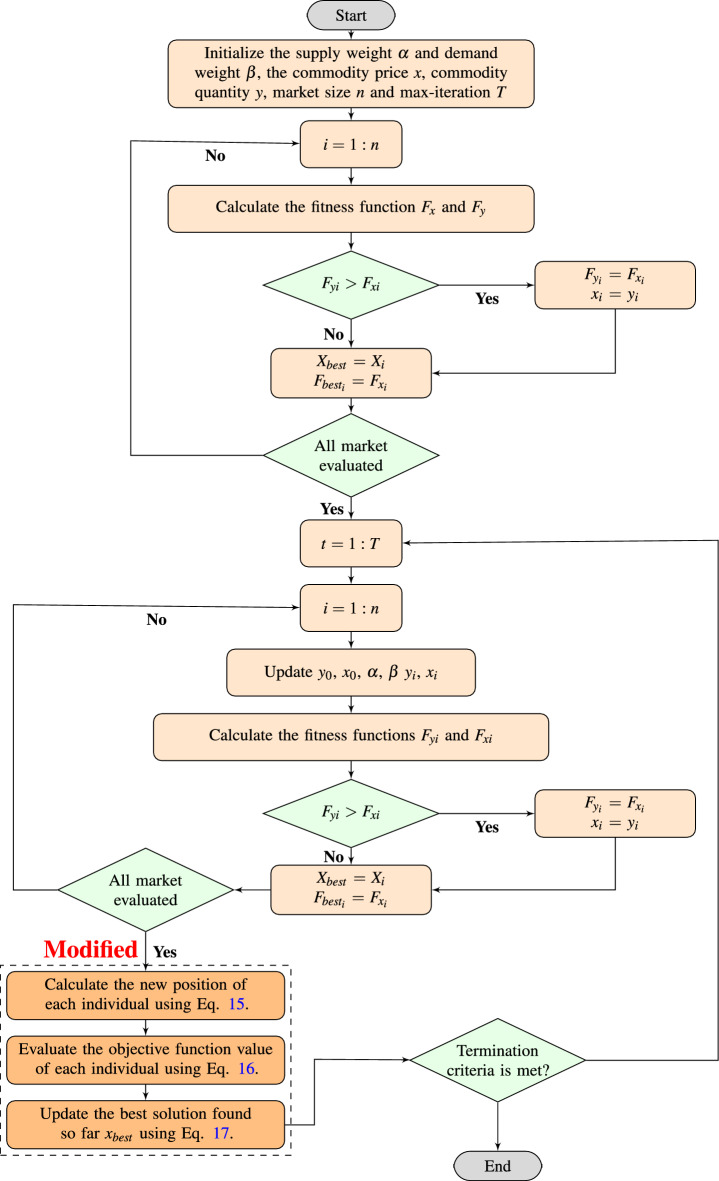
Flowchart of the proposed LSDO algorithm.

The diagram in Fig. 1 illustrates the flowchart of the Leader supply-demand-based optimization (LSDO) algorithm. It also depicts the position of Leader-based mutation selection in the algorithm. This modification has been incorporated to improve the exploration capability of the LSDO algorithm by performing simultaneous crossover and mutation using the three best leaders.

### Constraint handling superiority of feasible solutions (SF)

It is worth noting that the majority of optimization problems have both equality and inequality constraints that must be handled. However, almost all stochastic algorithms are unconstrained approaches. thereby, researchers process by employing the well-known static penalty strategy that is not reliable and requires control parameter settings. Along these lines, a superiority of feasible solutions (SF) constraint handling method is integrated into this study to deal with the constraints on state variables. Deb^50^ proposed the use of the Dominance-based approach for handling constraints, known as the SF strategy. This strategy is based on the concept of a dominant relationship, which gives priority to feasible solutions over infeasible ones. According to this strategy, a feasible candidate can always dominate an infeasible one, and a candidate with a smaller violation degree dominates the one with a higher violation value. The SF strategy employs a tournament selection operator, where two solutions are compared at a time. The solution $X_i$ is considered superior to $X_j$ if:

- An infeasible solution $X_j$ is dominated by a feasible one $X_i$
- if both $X_i$, $X_j$ are feasible, but $X_j$ is worst than $X_i$
- if both $X_i$, $X_j$ are infeasible, and $X_j$ has the greatest constraint violation.

The equality constraints are transformed into inequality constraints, resulting in the introduction of a total constraint as:

18 $$\begin{aligned} H_{i}(X)=\left\{ \begin{array}{l} \max \left( h_{i}(X), 0\right) \\ \max \left( |g_{i}(X) |-\delta , 0\right) \end{array}\right. \end{aligned}$$

where $\delta$ is a tolerance parameter for the equality constraints, $H_{i}(X)$ represents the inequality constraints. The expression of the constraint violation for an infeasible solution can be represented as:

19 $$\begin{aligned} V(X)=\frac{\sum \limits _{i=1}^{g} w_{i}\left( H_{i}(X)\right) }{\sum \limits _{i=1}^{g} w_{i}},\,\,\,\,\,\,w_{i} = \frac{1}{H_{max,i}} \end{aligned}$$

where $w_{i}$ is a weight factor, $H_{max,i}$ is the maximum value for violation of constraint.

## Problem formulation methodology

### Renewable energy model

**Figure 2:**
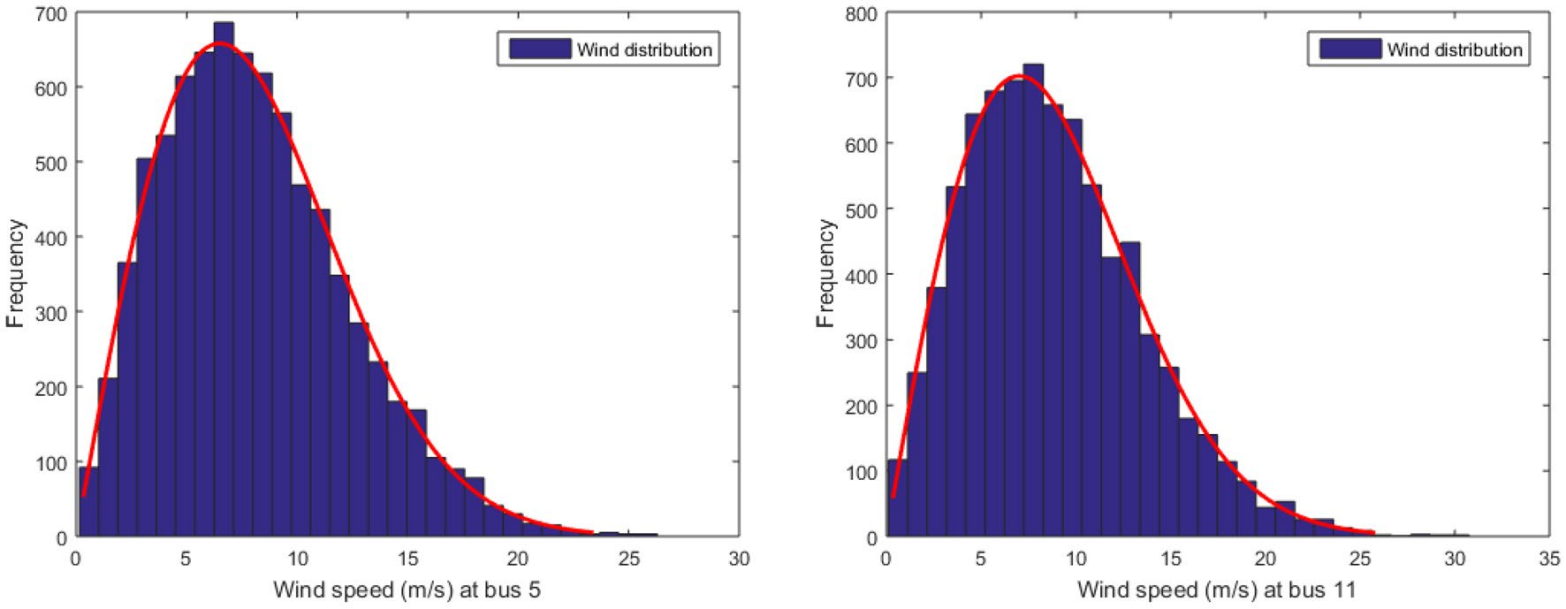
Distribution of wind speed for wind generators.

**Figure 3:**
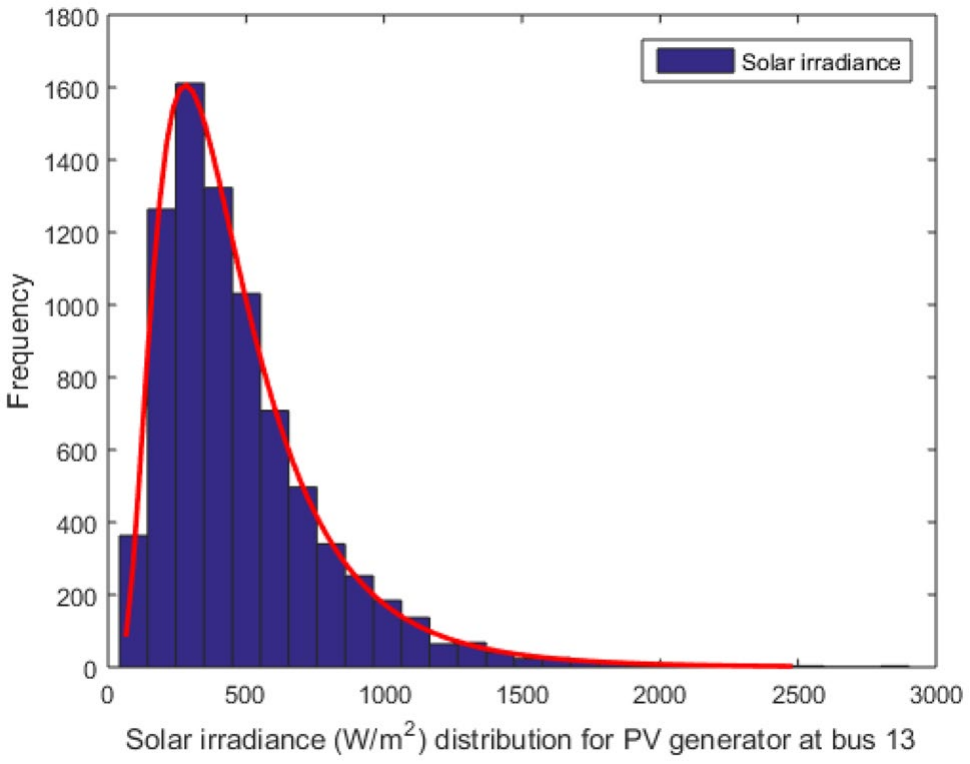
Distribution of solar irradiance for solar generator at 13th buses.

Presently, the integration of renewable energy resources (RESs) into power systems is rapidly advancing, with particular focus on wind and PV power. These RESs play a pivotal role in reducing CO2 emissions and bolstering the power system’s overall quality and reliability. To model solar irradiance and wind distribution, Lognormal and Weibull probability density functions are respectively utilized^51^. Through 8000 iterations of the Monte Carlo simulation, the Lognormal fitting of solar irradiance, Weibull fitting of wind speed, and Frequency distribution are obtained and visualized in Figs. 2, 3^52^. Each of these resources is associated with three cost components: direct cost, penalty cost, and reserve cost^51^. Table 1 provides a comprehensive description of all the parameters related to solar and wind energy sources.

**Table 1:** Characteristic details of wind-solar generators.

Test systems	Wind power				PV power		
	Wind	No of turbines	Pwr (MW)	Parameters of Weibull PDF	Solar	Psr (MW)	Parameters of Lognormal PDF
IEEE-30	1(bus 5)	25	75	k = 2, c = 9	(bus 13)	50	$\mu$ = 6, $\sigma$ = 0.6
	2(bus 11)	20	60	k = 2, c = 10			
IEEE-57	1(bus 2)	50	150	k = 2, c = 10	(bus 9)	50	$\mu$ = 6, $\sigma$ = 0.6
	2(bus 6)	40	120	k = 2, c = 10			

#### Wind power

The variability of wind flow is modeled using a Weibull probability distribution function^53^.

20 $$\begin{aligned} f(v)=\left( \frac{k}{c}\right) \left( \frac{v}{c}\right) ^{(k-1)} \exp \left[ -\left( \frac{v}{c}\right) ^{k}\right] , \quad v \ge 0 \end{aligned}$$

where the parameters *k* and *c* represent the shape and scale factors of the Weibull distribution, respectively.

The wind generator’s output power is determined by the stochastic wind speed and can be expressed as follows^53^:

21 $$\begin{aligned} \begin{array}{l} {p_{w}(v)=\left\{ \begin{array}{ll} {0} &{} {v<v_{\text{ in } } \text{ and } v>v_{\text{ out } }} \\ {p_{wr}\left( \frac{v-v_{\text{ in } }}{v_{r}-v_{\text{ in } }}\right) } &{} { v_{\text{ in } } \le v \le v_{r}} \\ {p_{wr}} &{} {v_{r}<v \le v_{\text{ out } }} \end{array}\right. } \end{array} \end{aligned}$$

where $v_{out}$, $v_{in}$, $v_{r}$, *v* and $p_{wr}$ are cut-out wind speed, cut-in wind speed, rated wind speed, actual wind speed, and rated output power, respectively.

The total cost of wind energy encompasses the following components^51^: direct Cost associated with the scheduled power generated by the wind turbine, penalty Cost of Underestimation, and reserve Cost for Overestimation. These factors together contribute to determining the overall cost associated with wind energy generation as represented below:

22 $$\begin{aligned} C_{T w, i w}=C_{d w, i w}+C_{u e w, i w}+C_{o e w, i w} \end{aligned}$$

with,

23 $$\begin{aligned} C_{d w, i}= & {} d_{w, i} P_{w s, i} \end{aligned}$$

24 $$\begin{aligned} C_{u e w, i}= & {} K_{u e w, i} \int \limits _{P_{w s, i}}^{P_{w r, i}}\left( p_{w, i}-P_{w s, i}\right) f_{w}\left( p_{w, i}\right) d p_{w, i} \end{aligned}$$

25 $$\begin{aligned} C_{o e w, i}= & {} K_{o e w, i} \int \limits _{0}^{P_{w s, i}}\left( P_{w s, i}-p_{w, i}\right) f_{w}\left( p_{w, i}\right) d p_{w, i} \end{aligned}$$

where $d_{w, i}$ is the coefficient of direct cost of *i*th wind generator. $K_{o e w, i}$ and $K_{u e w, i}$ are the over and under estimation cost coefficients pertaining to *i*th wind power plant. $p_{ws,i}$ is the scheduled power. $f_{w}\left( p_{w, i}\right)$ is the probability density function of *i*th wind power plant.

#### Solar power

The lognormal distribution is employed as the probability distribution function to calculate the PV output power, as illustrated below^53^:

26 $$\begin{aligned} f(G)=\frac{1}{G \sigma \sqrt{2 \pi }} \exp \left[ \frac{-(\ln x-\mu )^{2}}{2 \sigma ^{2}}\right] , \quad G \succ 0 \end{aligned}$$

The available power $P_s(G)$ of solar irradiation *G* is calculated in the following manner, as shown in^53^:

27 $$\begin{aligned} P_{s}(G)=\left\{ \begin{aligned} P_{s r}\left( \frac{G^{2}}{G_{std} R_{c}}\right)&0 \prec G \prec R_{c} \\ P_{s r}\left( \frac{G}{G_{s t d}}\right)&R_{c} \le G \end{aligned}\right. \end{aligned}$$

where $P_{s r}$, $G_{std}$, *G*, and $R_{c}$ are the rated output power of solar PV, solar irradiation in standard environment, forecasted solar irradiation, and certain irradiance point, respectively.

The PVs total cost is formulated as follows^51^:

28 $$\begin{aligned} C_{T s, i s}=C_{d s, i s}+C_{u e s, i s}+C_{o e s, i s} \end{aligned}$$

with,

29 $$\begin{aligned} C_{d s, i}= & {} d_{s, i} P_{s s, i} \end{aligned}$$

30 $$\begin{aligned} C_{u e s, i}= & {} K_{u e s, i} \int _{P_{s s, i}}^{P_{s r, i}}\left( p_{s, i}-P_{s s, i}\right) f_{s}\left( p_{s, i}\right) d p_{s, i} \end{aligned}$$

31 $$\begin{aligned} C_{o e s, i}= & {} K_{o e s, i} \int _{0}^{P_{s s, i}}\left( P_{s s, i}-p_{s, i}\right) f_{s}\left( p_{s, i}\right) d p_{s, i} \end{aligned}$$

where $d_{s, i}$ is the coefficient of direct cost of *i*th wind generator. $P_{s s, i}$ is the scheduled power. $K_{o e s, i}$ and $K_{u e s, i}$ are the over and under estimation cost coefficients of solar power plant. $f_{s}\left( p_{s, i}\right)$ is the probability density function of the *i*th solar power plant.

### Optimal power flow model

Generally speaking, OPF is considered a complex, non-convex, non-linear in power system optimization problem. The purpose of OPF is to minimize various competing objective functions subject to diverse control and state variables, as well as power flow equations and unit operating limits as equality and inequality constraints, respectively.

#### Objective functions

In this work, six competing objective functions will be outlined.

##### Fuel cost

 The total fuel cost of the network’s generators is modeled as a quadratic function, expressed as follows^51^:

32 $$\begin{aligned} F_{1}= F_{c}(s,c)=min\left\{ \begin{array}{ll} \sum _{i=1}^{Ng}a_i+b_iP_{gi}+c_iP_{gi}^2 \\ \end{array} \right. \end{aligned}$$

where $a_i$, $b_i$ and $c_i$ are the cost coefficients of the conventional units.

##### Emission

 The emission function is represented using an exponential function that is formulated based on the previous quadratic function as follows^51^:

33 $$\begin{aligned} F_{2}= E(s,c)=min\left\{ \sum _{i=1}^{N g} 10^{-2} \left( \alpha _{i}+\beta _{i} P_{g i}+\gamma _{i} P_{g i}^{2}\right) +\xi _{i} \exp \left( \lambda _{i} P_{g i} \right) \right. \end{aligned}$$

where $\alpha _{i}$, $\beta _{i}$, $\gamma _{i}$, $\xi _{i}$, and $\lambda _{i}$ are the emission coefficients of the power plant.

##### Voltage deviation

 The load bus voltages are set to 1.0 per unit to ensure a desirable voltage profile. The voltage deviation is defined as follows^54^:

34 $$\begin{aligned} F_{3}= VD(s,c) =min\left\{ \begin{array}{ll} \sum \limits _{i=1}^{Npq}|V_{Li}-1.0|\\ \end{array} \right. \end{aligned}$$

##### Power loss

 The transmission system experiences power losses due to the inherent resistance of the transmission lines. This can be mathematically modeled using the following expression^54^:

35 $$\begin{aligned} F_{4}= P_{loss}(s,c) = min\left\{ \begin{array}{ll} \sum \limits _{l=1}^{N_{l}}G_{l(i,j)}(V_i^2+V_j^2-2V_iV_jcos(\delta _{ij})) \end{array} \right. \end{aligned}$$

where, $G_{l(i,j)}$ represents the conductance of line *l*. $\delta _{ij} = \delta _i - \delta _j$ represents the voltage angle difference between bus *i* and bus *j*.

##### Cost considering renewable energy powers

 The total cost of the network, considering the combined contributions of wind, solar, and thermal powers, is expressed as follows^51^.

36 $$\begin{aligned} F_{5}= F_T(s,c) = min\left\{ F_{c} + C_{Tw} + C_{Ts} \right. \end{aligned}$$

where $F_{c}$, $C_{Tw}$, and $C_{Ts}$ are fuel cost, wind’s total cost, and PV’s total cost, respectively.

##### Cost considering renewable energy powers with the carbon tax

 Over the past decade, numerous countries have responded to global environmental concerns by introducing carbon taxes as a measure to mitigate carbon emissions into the environment. The calculation of emissions cost ($/ton) involves the application of a carbon tax ($$C_{Tax}$$) on emitted pollutants^51^:

37 $$\begin{aligned} F_{6} = min\left\{ F_{T} + E_{c} \right. \end{aligned}$$

avec

38 $$\begin{aligned} E_{c} = C_{Tax} \cdot E \end{aligned}$$

where *E* presents the emission, and $C_{Tax}=20$.

#### Variables

The set of state variables *s* can be defined as^51^:

39 $$\begin{aligned} s=[P_{g1},V_{L1},...,V_{LNpq},Q_{g1},...,Q_{gNg},S_{l1},...,S_{lNl}] \end{aligned}$$

where, $P_{g1}$ is the active power output at the slack bus. $V_L$ is the voltage magnitude at *PQ* buses. $Q_g$ is the reactive power output of all generator units. $S_l$ is the transmission line loading (line flow). $N_{pq}$, $N_g$, and $N_l$ denote the number of load buses, number of generating units, and number of transmission lines, respectively.

The set of control variables *c* can be expressed as^51^:

40 $$\begin{aligned} c=[P_{g2},...,P_{gNg},V_{g1},...,V_{gNg},Q_{c1},...,Q_{cNc},T_1,...,T_{NT}] \end{aligned}$$

The expression represents the modeling of transmission system power losses, which occur due to the resistance of lines. The active power generation at the *PV* buses, except the slack bus, is denoted by $P_g$, and $V_g$ represents the voltage magnitude at *PV* buses. The transformer tap settings are represented by *T*, and $Q_c$ is the shunt *VAR* compensation. $N_g$, $N_c$, and $N_T$ are the number of generators, regulating transformers, and *VAR* compensators (shunt), respectively.

#### Constraints

As previously mentioned, the OPF problem comprises both equality and inequality constraints, which are crucial in optimal power flow investigations as they represent the physical limitations of the equipment. The constraints are modeled as follows:

*Equality constraints* The power flow equations are assumed as equality constraints that are represented by:

41 $$\begin{aligned} \left\{ \begin{array}{l} P_{gi}-P_{di}-|V_i|\sum _{j=1}^{Nb}|V_j|[G_{ij}cos(\theta _{ij})+B_{ij}sin(\theta _{ij})]=0 \\ Q_{gi}-Q_{di}-|V_i|\sum _{j=1}^{Nb}|V_j|[G_{ij}sin(\theta _{ij}){-}B_{ij}cos(\theta _{ij})]=0 \end{array}\right. \end{aligned}$$

The number of buses in the system is denoted by *Nb*. The active and reactive power generated at bus *i* are represented by $P_{gi}$ and $Q_{gi}$, respectively, while the active and reactive power demand at bus *i* are represented by $P_{di}$ and $Q_{di}$, respectively. The admittance matrix components are denoted by $G_{ij}$ and $B_{ij}$. $Y_{ij}=G_{ij}+jB_{ij}$ named the conductance and susceptance.

*Inequality constraints* The inequality constraints are given below:

- Generator constraints:

42 $$\begin{aligned}{} & {} V_{gi}^{min}\le V_{gi}\le V_{gi}^{max}\,\,\,\,\,\,\,\,\,\,\,\,\,i=1,...,Ng \end{aligned}$$

43 $$\begin{aligned}{} & {} P_{gi}^{min}\le P_{gi}\le P_{gi}^{max}\,\,\,\,\,\,\,\,\,\,\,\,\,\,i=1,...,Ng \end{aligned}$$

44 $$\begin{aligned}{} & {} P_{ws,i}^{min}\le P_{ws,i}\le P_{ws,i}^{max}\,\,\,\,\,\,\,\,\,\,\,\,\,\,i=1,...,Nwg \end{aligned}$$

45 $$\begin{aligned}{} & {} P_{ss,i}^{min}\le P_{ss,i}\le P_{ss,i}^{max}\,\,\,\,\,\,\,\,\,\,\,\,\,\,i=1,...,Nsg \end{aligned}$$

46 $$\begin{aligned}{} & {} Q_{gi}^{min}\le Q_{gi}\le Q_{gi}^{max}\,\,\,\,\,\,\,\,\,\,\,\,i=1,...,Ng \end{aligned}$$

47 $$\begin{aligned}{} & {} Q_{ws,i}^{min}\le Q_{ws,i}\le Q_{ws,i}^{max}\,\,\,\,\,\,\,\,\,\,\,\,i=1,...,Nwg \end{aligned}$$

48 $$\begin{aligned}{} & {} Q_{ss,i}^{min}\le Q_{ss,i}\le Q_{ss,i}^{max}\,\,\,\,\,\,\,\,\,\,\,\,i=1,...,Nsg \end{aligned}$$

where $V_i^{min}$ and $V_i^{max}$ indicate the minimum and maximum bounds of the bus voltage. $P_{gi}^{min}$ and $P_{gi}^{max}$ represent the lower and upper bounds of active power generators. $Q_{gi}^{min}$ and $Q_{gi}^{max}$ are the minimum and maximum reactive power bounds of the generator. $P_{ws,i}^{min}$, $P_{ws,i}^{max}$, $P_{ss,i}^{min}$, $P_{ss,i}^{max}$, $Q_{ws,i}^{min}$, $Q_{ws,i}^{max}$, $Q_{ss,i}^{min}$, and $Q_{ss,i}^{max}$ are the bounds of energy resources. *Ng*, *Nwg*, and *Nsg* are the number of generation, wind, and solar, respectively.

- Transformer constraints:

49 $$\begin{aligned} T_i^{min}\le T_i\le T_i^{max}\,\,\,\,\,\,\,\,\,\,\,\,\,\,\,\,i=1,...,N_T \end{aligned}$$

where, $N_T$ is the number of tap changer transformers. $T_i^{min}$ and $T_i^{max}$ represent the minimum and maximum limits of the transformer, respectively.

- Shunt VAR compensators constraints:

50 $$\begin{aligned} Q_{ci}^{min}\le Q_{ci}\le Q_{ci}^{max}\,\,\,\,\,\,\,\,\,\,\,\,\,\,i=1,...,Nc \end{aligned}$$

where, *Nc* is the number of capacitor components. $Q_{c,i}^{min}$ and $Q_{c,i}^{max}$ are the minimum and maximum limits of the shunt compensators.

- Security constraints:

51 $$\begin{aligned}{} & {} V_{Li}^{min}\le V_{Li}\le V_{Li}^{max}\,\,\,\,\,\,\,\,\,\,\,\,\,i=1,...,Npq \end{aligned}$$

52 $$\begin{aligned}{} & {} S_{li}\le S_{li}^{max}\,\,\,\,\,\,\,\,\,\,\,\,\,\,\,\,\,\,\,\,\,\,\,\,\,\,i=1,...,Nl \end{aligned}$$

where, *Nl* is the number of transmission lines. $S_{li}$ and $S_{li}^{max}$ indicate the maximum limit of the transmission line.

## Simulation results and discussion

This section demonstrates the superiority of the proposed LSDO algorithm through experimentation with 23 benchmark functions. All 23 experiments were conducted using MATLAB (R2020a) on a computer with an Intel(R) Core(TM) i5-9400F CPU running at 2.90 GHz and 8GB of RAM.

### Simulation results of benchmark functions

In this subsection, the effectiveness and accuracy of the LSDO technique are evaluated using 23 benchmark functions^55^. These functions are divided into three categories: uni-modal functions (F1–F7), multi-modal functions (F8–F14), and fixed-dimension multi-modal functions. Table 2 provides the definitions of these functions, with D, UM, and MM representing the dimension, uni-modal functions, and multi-modal functions, respectively. The performance of the original SDO technique and three well-known optimization algorithms, namely social network search (SNS)^56^, gray wolf optimizer (GWO)^57^, and tunicate swarm algorithm (TSA)^58^, are also compared. The evaluation metrics include the best, mean, median, worst values, and standard deviation (std) of the solutions obtained by each algorithm. Table 3 presents the results, where all algorithms were run with a population size of 50 and a maximum of 200 iterations for 20 independent runs. As shown, the proposed LSDO technique achieves the best values for most benchmark functions.

**Table 2:** Definition of 23 benchmark functions.

No	Name	D	Range	Type	$f_{\min }$
F1	Sphere	[30, 100]	$[-100,100]$	UM	0
F2	Schwefel 2.22	[30, 100]	$[-10,10]$	UM	0
F3	Schwefel 1.2	[30, 100]	$[-100,100]$	UM	0
F4	Schwefel 2.21	[30, 100]	$[-100,100]$	UM	0
F5	Rosenbrock	[30, 100]	$[-30,30]$	UM	0
F6	Step	[30, 100]	$[-100,100]$	UM	0
F7	Quartic	[30, 100]	$[-1.28,1.28]$	UM	0
F8	Schwefel	[30, 100]	$[-500,500]$	$\textrm{MM}$	$-12,569.487$
F9	Rastrigin	[30, 100]	$[-5.12,5.12]$	MM	0
F10	Ackley	[30, 100]	$[-32,32]$	MM	0
F11	Griewank	[30, 100]	$[-600,600]$	MM	0
F12	Penalized	[30, 100]	$[-50,50]$	MM	0
F13	Penalized 2	[30, 100]	$[-50,50]$	$\textrm{MM}$	0
$\textrm{F} 14$	Foxholes	2	$$\begin{array}{c}{[-65.536,} \\ 65.536]\end{array}$$	$\textrm{MM}$	0.998004
F15	Kowalik	4	$[-5,-5]$	MM	0.0003075
F16	Six-hump	2	$[-5,-5]$	$\textrm{MM}$	−1.0316285
F17	Branin	2	$[-5,-5]$	MM	0.398
F18	$$\begin{array}{l} \text{ Goldstein- } \\ \text{ Price } \end{array}$$	2	$[-2,2]$	$\textrm{MM}$	3
F19	Hartman 3	3	$[-1,2]$	MM	−3.862782
F20	Hartman 6	6	[0, 1]	MM	−3.32236
F21	Shekel 5	4	[0, 10]	MM	−10.1532
F22	Shekel 7	4	[0, 10]	MM	−10.4029
F23	Shekel 10	4	[0, 10]	MM	−10.5364

**Table 3:** Statistical results of 23 benchmark functions by the proposed LSDO technique and other recent algorithms.

Function		LSDO	SDO	TSA	GWO	SNS
F1	Min	**8.3E−151**	1.39E−55	3.79E−08	4.47E−12	1.03E−28
	Average	**4.6E−128**	1.37E−51	3.92E−07	3.12E−11	1.37E−27
	Median	**4.4E−140**	3.74E−54	1E−07	2.46E−11	4.77E−28
	Max	**9.1E−127**	8.43E−51	4.09E−06	8.73E−11	1.04E−26
	Std	**2E−127**	2.74E−51	9.2E−07	2.31E−11	2.38E−27
F2	Min	**1.01E−69**	1.83E−29	2.44E−06	1.42E−07	2.3E−15
	Average	**2E−60**	3.76E−25	1.9E−05	2.77E−07	5.64E−15
	Median	**6.56E−63**	1.13E−26	1.86E−05	2.66E−07	4.21E−15
	Max	**2.53E−59**	3.98E−24	3.68E−05	4.78E−07	1.4E−14
	Std	**5.95E−60**	9.1E−25	9.44E−06	9.9E−08	3.51E−15
F3	Min	**2.2E−145**	6.27E−46	0.027608	0.008462	9.18E−13
	Average	**8.7E−120**	6.91E−34	1.122677	0.610441	4.18E−08
	Median	**9.3E−138**	1.4E−39	0.772195	0.185412	4.13E−09
	Max	**1.7E−118**	1.38E−32	3.914695	3.567009	3.9E−07
	Std	**3.9E−119**	3.09E−33	1.096313	0.827115	9.17E−08
F4	Min	**4.81E−73**	1.11E−26	0.67531	0.002608	1.33E−13
	Average	**1.63E−67**	4.52E−23	3.616654	0.008	5.45E−13
	Median	**5.11E−70**	1.14E−23	3.022253	0.007092	4.09E−13
	Max	**1.67E−66**	1.94E−22	9.361516	0.016667	1.87E−12
	Std	**4.02E−67**	6.34E−23	2.343658	0.003845	4.55E−13
F5	Min	26.36703	27.90967	27.18973	**25.92515**	27.6644
	Average	**26.99756**	28.65096	39.01094	27.18903	28.03399
	Median	**26.86409**	28.74726	28.66203	27.09814	27.97984
	Max	28.66131	28.98699	239.7785	28.79035	**28.44604**
	Std	0.508238	0.295026	47.26339	0.72182	**0.216873**
F6	Min	**0.020726**	0.039957	2.886997	0.252254	0.080879
	Average	**0.141345**	2.568541	3.800719	0.647554	0.292241
	Median	**0.082029**	2.038779	3.736935	0.611378	0.255115
	Max	**0.549917**	7.250251	4.850371	1.172757	0.75842
	Std	**0.154727**	1.852701	0.527851	0.280888	0.181696
F7	Min	**3.93E−05**	8.66E−05	0.007604	0.001477	0.000168
	Average	**0.000179**	0.002356	0.019206	0.004433	0.000708
	Median	**0.000139**	0.001136	0.018479	0.003685	0.000688
	Max	**0.000513**	0.013813	0.04436	0.01033	0.002187
	Std	**0.000146**	0.003331	0.007628	0.002554	0.000488
F8	Min	−1733.12	−1655	−1394.45	−1495.31	**−7613.49**
	Average	−1534.59	−1312.83	−1212.82	−1245.57	**−6358.62**
	Median	−1536.16	−1385.86	−1232.52	−1224.18	**−6324.46**
	Max	−1327.7	−598.802	−976.635	−1123.85	**−5562.96**
	Std	**94.79719**	294.008	122.0762	104.0153	538.2484
F9	Min	**0.00**	4.33E−30	156.667	1.062467	**0.00**
	Average	**0.00**	1.75E−22	228.0177	9.801018	**0.00**
	Median	**0.00**	4.17E−25	228.634	9.824713	**0.00**
	Max	**0.00**	3.02E−21	331.7581	24.96968	**0.00**
	Std	**0.00**	6.75E−22	46.40919	5.565812	**0.00**
F10	Min	**8.88E−16**	**8.88E−16**	20.81133	20.76487	4.44E−15
	Average	**8.88E−16**	**8.88E−16**	20.9608	20.92344	7.46E−15
	Median	**8.88E−16**	**8.88E−16**	20.99356	20.94465	6.22E−15
	Max	**8.88E−16**	**8.88E−16**	21.0961	21.06309	1.51E−14
	Std	**0.00**	**0.00**	0.091505	0.083433	3.69E−15
F11	Min	**0.00**	**0.00**	1.3E−09	6.56E−13	**0.00**
	Average	**0.00**	**0.00**	0.007018	0.009891	**0.00**
	Median	**0.00**	**0.00**	1.44E−08	4.55E−12	**0.00**
	Max	**0.00**	**0.00**	0.029126	0.055407	**0.00**
	Std	**0.00**	**0.00**	0.010243	0.015766	**0.00**
F12	Min	**0.000176**	0.001152	0.374956	0.006066	0.000696
	Average	**0.003854**	0.23467	2.805889	0.026151	0.00268
	Median	**0.002177**	0.067805	2.009833	0.023474	0.00284
	Max	0.01893	1.492821	7.656863	0.047176	**0.004893**
	Std	0.004455	0.352063	2.128936	0.013414	**0.001232**
F13	Min	0.175696	**0.046216**	2.372295	0.09955	0.057519
	Average	0.834851	1.867552	3.298085	**0.613832**	0.154385
	Median	**0.451512**	1.934246	3.22876	0.609981	0.140323
	Max	2.966605	2.999924	4.16073	**1.044**	0.378672
	Std	0.957067	0.961284	0.565835	**0.280029**	0.077659
F14	Min	**0.998004**	**0.998004**	**0.998004**	**0.998004**	**0.998004**
	Average	**0.998004**	3.494696	8.298683	3.892106	**0.998004**
	Median	**0.998004**	1.495017	10.76318	2.982105	**0.998004**
	Max	**0.998004**	**12.67051**	18.30431	**12.67051**	**0.998004**
	Std	**0.00**	3.953203	5.533952	3.727681	1.02E−16
F15	Min	**0.000307**	**0.000307**	0.000308	0.00031	0.000308
	Average	**0.000308**	0.00067	0.007136	0.003547	0.00035
	Median	**0.000307**	0.000527	0.000505	0.000546	0.000313
	Max	**0.000309**	0.002121	0.031699	0.020363	0.000582
	Std	**3.22E−07**	0.000473	0.010606	0.007255	6.8E−05
F16	Min	**−1.03163**	**−1.03163**	**−1.03163**	**−1.03163**	**−1.03163**
	Average	**−1.03163**	−1.03005	−1.0253	**−1.03158**	**−1.03163**
	Median	**−1.03163**	**−1.03163**	**−1.03163**	**−1.03163**	**−1.03163**
	Max	**−1.03163**	−1.00046	−0.99999	**−1.03063**	**−1.03163**
	Std	2.28E−16	0.006966	0.012981	0.000223	**1.53E−16**
F17	Min	**0.397887**	**0.397887**	0.39789	0.397888	**0.397887**
	Average	**0.397887**	**0.397987**	0.397927	0.397891	**0.397887**
	Median	**0.397887**	**0.397887**	0.397907	0.397891	**0.397887**
	Max	**0.397887**	0.399795	0.398082	**0.397897**	**0.397887**
	Std	**0.00**	0.000426	4.53E−05	3.01E−06	**0.00**
F18	Min	**0.00**	**3.00**	3.000009	**3.00**	**0.00**
	Average	**0.00**	**3.00**	8.400078	3.000068	**0.00**
	Median	**0.00**	**3.00**	3.000084	3.000036	**0.00**
	Max	**0.00**	**3.00**	84.00001	3.000238	**0.00**
	Std	**5.49E−16**	5.21E−08	18.78799	6.53E−05	1.6E−15
F19	Min	−0.30048	−0.30048	−0.30048	−0.30048	**−3.86278**
	Average	−0.30048	−0.2893	−0.30048	−0.30048	**−3.86278**
	Median	−0.30048	−0.30038	−0.30048	−0.30048	**−3.86278**
	Max	−0.30048	−0.19165	−0.30048	−0.30048	**−3.86278**
	Std	**1.14E−16**	0.026531	**1.14E−16**	**1.14E−16**	2.22E−15
F20	Min	**−3.322**	**−3.322**	−3.32148	−3.32198	**−3.322**
	Average	−3.23283	−3.09697	−3.07223	−3.22876	**−3.29822**
	Median	−3.2031	−3.2031	−3.20118	−3.26239	**−3.322**
	Max	−3.2031	−0.89904	−0.20816	−2.84039	**−3.2031**
	Std	0.05282	0.550986	0.679321	0.125558	**0.048793**
F21	Min	**−10.1532**	**−10.1532**	−10.0895	−10.1502	**−10.1532**
	Average	**−10.1532**	−8.703	−5.89545	−8.51218	**−10.1532**
	Median	**−10.1532**	**−10.1532**	−4.90994	−10.1413	**−10.1532**
	Max	**−10.1532**	−4.99677	−2.58642	−2.62918	**−10.1532**
	Std	**3.36E−15**	2.23952	2.775111	2.963153	2.8E−12
F22	Min	**−10.4029**	**−10.4029**	−10.3637	−10.4024	**−10.4029**
	Average	**−10.4029**	−8.45822	−7.02119	**−10.0134**	**−10.4029**
	Median	**−10.4029**	**−10.4029**	−9.8942	−10.3959	**−10.4029**
	Max	**−10.4029**	−1.0677	−1.82478	−2.76526	**−10.4029**
	Std	**3.13E−15**	3.128689	3.57071	**1.706042**	5.02E−15
F23	Best	**−10.5364**	**−10.5364**	−10.4599	−10.5348	**−10.5364**
	Mean	−10.266	−7.90449	−5.50502	**−9.74305**	**−10.5364**
	Median	**−10.5364**	−10.5357	−2.83596	−10.5274	**−10.5364**
	Worst	−5.12848	−3.79083	−1.66783	−2.42135	**−10.5364**
	Std	1.20925	3.015319	3.728197	2.418464	**2E−15**

The best values obtained are in bold.

**Figure 4:**
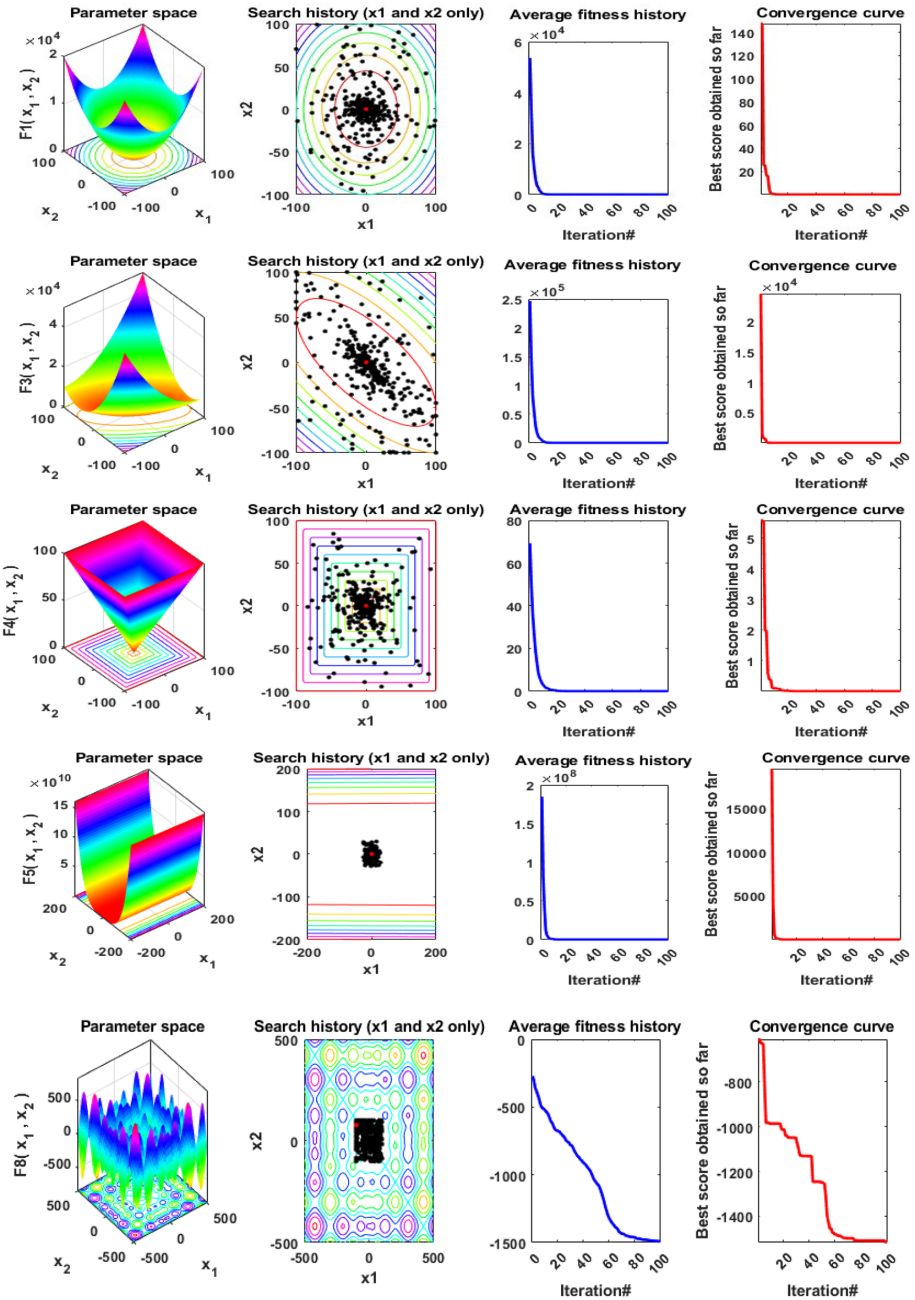

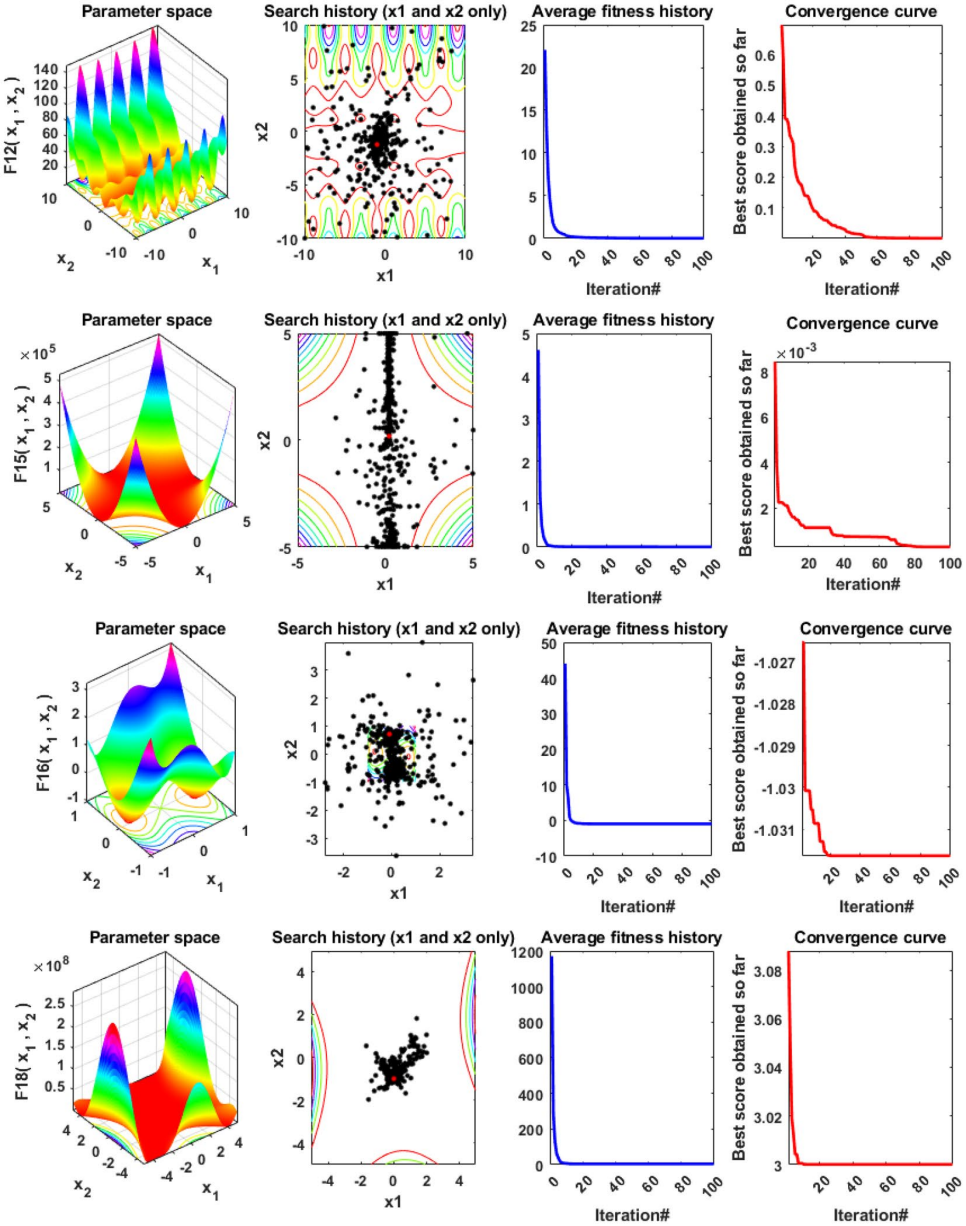
Qualitative metrics of nine benchmark functions: 2D views of the functions, search history, average fitness history, and convergence curve using the proposed LSDO technique.

**Figure 5:**
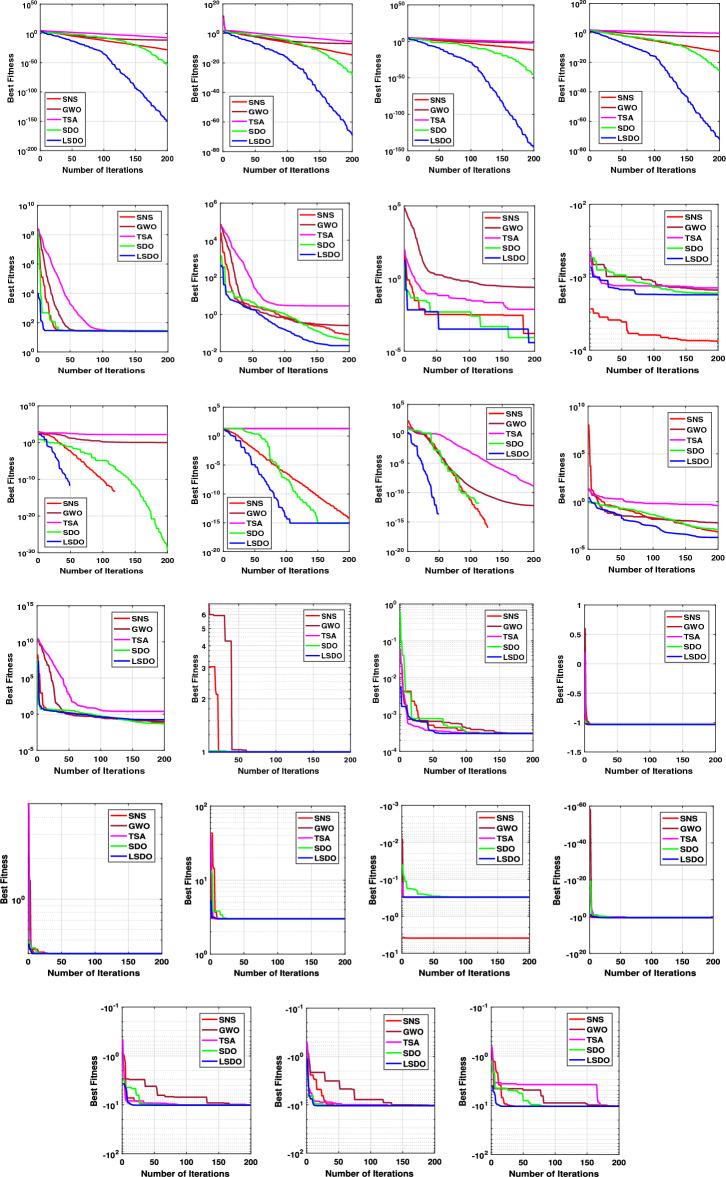
The convergence curves of studied algorithms for 23 benchmark functions.

**Figure 6:**
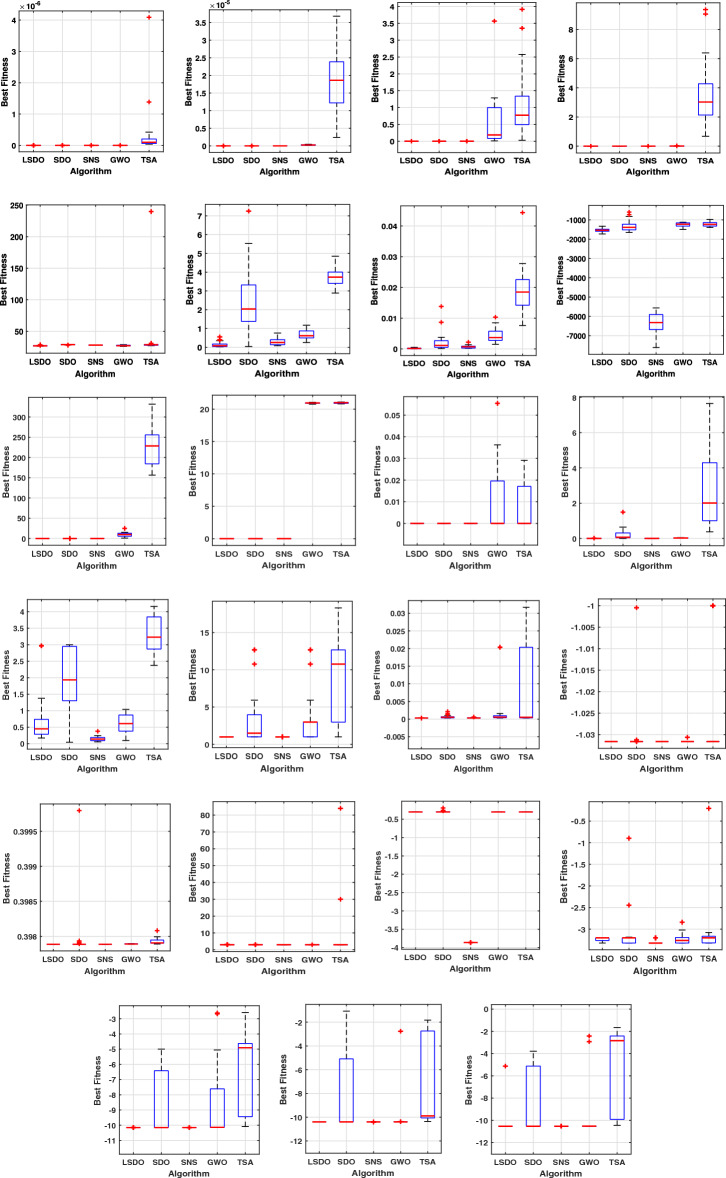
Boxplots of studied algorithms for 23 benchmark functions.

In addition, qualitative metrics of the proposed LSDO technique for nine benchmark functions are shown in Fig. 4, including 2D views of the functions, search history, average fitness history, and convergence curves. The convergence curves for all algorithms and benchmark functions are illustrated in Fig. 5, while the boxplots are displayed in Fig. 6. The LSDO algorithm is observed to reach a stable point for all functions, and its boxplots are narrower than the other techniques for many functions.

The LSDO technique’s performance is compared to other recent algorithms including the original SDO technique and six well-known optimization algorithms, namely SNS, GWO, TSA, differential evolution (DE)^59^, particle swarm optimizer (PSO)^60^, and artificial bee colony (ABC)^61^ on 13 benchmark functions with a dimension of 100. The results are presented in Table 4. For Function 1, the proposed LSDO technique achieved significantly better results with a minimum value of 1.7E−145, outperforming other algorithms. Function 2 also demonstrated the superiority of the LSDO technique, as it obtained a minimum value of 3.77E−68, notably better than the other algorithms. The LSDO technique performed exceptionally well on Function 3, achieving a minimum value of 6.97E−143, which significantly outperformed other algorithms. Function 4 also showed the superiority of the LSDO technique with a minimum value of 2.06E−73, outclassing other algorithms in this benchmark. For Function 5, the LSDO technique yielded promising results with a minimum value of 96.87, while maintaining competitive performance with the other algorithms. Function 6 showcased the strength of the LSDO technique with a minimum value of 6.7327, outperforming other algorithms. In Function 7, the LSDO technique obtained an impressively low minimum value of 2.91E−06, significantly improving compared to other algorithms. The LSDO technique demonstrated its effectiveness in Function 8, achieving a minimum value of −4014.5, which is significantly better than the results obtained by other algorithms. Function 9 showcased the superiority of the LSDO technique, as it achieved a minimum value of 0, outperforming other algorithms. Function 10 also displayed the strength of the LSDO technique with a minimum value of 8.88E−16, demonstrating superior performance compared to other algorithms. The LSDO technique excelled in Function 11, achieving a minimum value of 0, and outperforming other algorithms. Function 12 showcased the effectiveness of the LSDO technique with a minimum value of 0.04123, displaying better results compared to other algorithms. In Function 13, the LSDO technique achieved an excellent minimum value of 5.7551, outclassing other algorithms. Overall, the LSDO technique consistently displayed superior performance in multiple benchmark functions, achieving the best results in most cases. These findings indicate the potential effectiveness and competitiveness of the proposed LSDO technique for solving optimization problems.

**Table 4:** Statistical results of 13 benchmark functions (Dim = 100) by the proposed LSDO technique and other recent algorithms.

Function		LSDO	SDO	TSA	GWO	SNS	DE	PSO	ABC
F1	Min	**1.7E−145**	1.5E−53	0.016	0.0005	1.7E−26	35997	198.4	250910
	Average	** 6.2E−129**	1.1E−49	0.0865	0.00188	4.39E−25	37253.8	269.9	264305.6
	Median	**1.5E−138**	1.25E−51	0.07846	0.00162	1.38E−25	36323.4	289.52	269156.2
	Max	**1.23E−127**	1.34E−48	0.2354	0.0037	4.12E−24	39025.8	369.43	269584.1
	Std	**2.7E−128**	3.15E−49	0.05368	0.00088	9.87E−25	1527.2	71.8248	8101.7
F2	Min	**3.77E−68**	5.78E−29	0.01247	0.00715	5.07E−14	171.25	8.823	1.37E+36
	Average	**2.28E−56**	2.995E−26	0.055	0.00988	1.95E−13	186.282	11.674	5.41E+40
	Median	**5.05E−59**	8.06E−27	0.0435	0.0097	1.36E−13	188.838	11.252	6.36E+37
	Max	**1.87E−55**	1.82E−25	0.1365	0.015	4.82E−13	194.866	17.426	2.5E+41
	Std	**4.87E−56**	4.89E−26	0.03697	0.00188	1.28E−13	9.2358	3.47125	1.12E+41
F3	Min	**6.97E−143**	4.16E−43	10404.1	1563.5	2.66E−05	360641.8	19800.8	891967.8
	Average	**9.13E−121**	6.54E−35	27016.3	7361.1	0.00075	502022.6	32155.39	1072174
	Median	**6.57E−132**	1.60E−38	20987.67	6745.44	0.000211	510654.9	37007.8	1047367
	Max	**1.82E−119**	1.26E−33	57599.01	17021.3	0.005016	588790.1	42029.19	1309319.6
	Std	**4.08E−120**	2.83E−34	12914.3	4420.7	0.00128	85313.2	9705.4	169362.2
F4	Min	**2.06E−73**	1.08E−26	46.4652	2.6979	3.1E−12	91.186	18.055	91.8624
	Average	**3.31E−65**	5.26E−24	65.0496	7.2693	1.11E−11	92.3478	22.8813	94.124
	Median	**1.54E−68**	1.53E−24	66.3633	7.03937	8.95E−12	92.637	22.178	94.44
	max	**2.48E−64**	2.45E−23	78.736	16.437	2.47E−11	93.1829	26.433	96.5098
	Std	**7.45E−65**	7.52E−24	9.1168	3.127	6.81E−12	0.76617	3.4285	1.79575
F5	Min	**96.87**	97.58	100.32	97.68	97.826	64005640	12405.4	11E+07
	Average	**97.887**	98.3307	117.264	98.818	98.3699	76664058	25776.446	12E+07
	Median	**98.115**	98.379	109.315	98.804	98.48	74721566	22752.86	11E+07
	Max	**98.44**	98.589	228.648	100.563	98.57	87181633	42821.401	13E+07
	Std	0.5151	0.2830	28.521	0.653	**0.2068**	9539385	11449.8	72615942
F6	Min	7.17914	**6.7327**	14.112	9.5161	9.2678	35136.3	271.043	255833.9
	Average	**8.7941**	11.487	15.60004	11.71104	10.699	37918.9	316.5084	266600
	Median	**8.8162**	11.767	15.664	11.8462	10.6814	38504.88	294.417	260500
	Max	**10.5304**	13.506	17.833	13.6589	12.089	39162.08	407.805	282601
	Std	**0.735**	1.7938	1.1548	1.2797	0.807	1627.52	57.454	12658.97
F7	Min	**2.91E−06**	9.33E−05	0.05508	0.01187	0.000204	101.809	0.39367	1643.169
	Average	**0.000314**	0.00042	0.1225	0.02078	0.00109	121.011	0.5299	1812.01
	Median	**0.00027**	0.00036	0.11843	0.01827	0.001006	118.666	0.54286	1806.14
	Max	**0.00086**	0.00118	0.20183	0.03927	0.002287	143.151	0.6279	2020.74
	Std	**0.00025**	0.00029	0.0327	0.0074	0.00059	15.237	0.09562	167.748
F8	Min	−4014.5	−4014.1	−3428.2	−3334.6	−1156.1	−3374.3	−4391.3	**−1.8E+26**
	Average	−3372.3	−3482.9	−2910.8	−2906.1	−803.2	−3246.6	−4064.9	**−4.5E+25**
	Median	−3342.1	−3560.4	−2929.1	−2851.5	−786.94	−3242.5	−4072.2	**−1.7E+25**
	Max	−2538.5	−2792.5	−2463.3	−2321.8	−437.4	−3149.6	−3785.3	**−6.3E+23**
	Std	374.029	295.14	203.94	261.40	175.47	83.857	263.346	**7.6E+25**
F9	Min	**0**	**0**	943.78	30.692	**0**	1248.01	321.17	3610.6
	Average	**0**	**0**	1186.25	74.059	**0**	1328.012	394.078	3731.58
	Median	**0**	**0**	1141.7	69.27	**0**	1354.3	397.8	3764.2
	Max	**0**	**0**	1547.84	124.25	**0**	1392.99	442.77	3788.51
	Std	**0**	**0**	164.597	21.866	**0**	61.881	50.832	71.380
F10	Min	**8.88E−16**	**8.88E−16**	21.233	21.23	4.8982	20.424	20.44	21.358
	Average	**8.88E−16**	**8.88E−16**	21.3008	21.2787	18.8026	20.439	20.713	21.390
	Median	**8.88E−16**	**8.88E−16**	21.297	21.285	20.0276	20.445	20.65	21.402
	Max	**8.88E−16**	**8.88E−16**	21.355	21.327	20.182	20.457	21.044	21.425
	Std	**0**	**0**	0.0307	0.03007	3.9447	0.01447	0.22407	0.0297
F11	Min	**0**	**0**	0.00025	1.25E−05	**0**	8.852	1.0257	64.195
	Average	**0**	**0**	0.0451	0.0104	**0**	9.5616	1.0672	68.988
	Median	**0**	**0**	0.0021	2.92E−05	**0**	9.560	1.06673	68.47
	Max	**0**	**0**	0.2698	0.06162	**0**	9.966	1.1216	72.342
	Std	**0**	**0**	0.06533	0.0215	**0**	0.454	0.0385	3.4515
F12	Best	0.10077	0.1015	2.034	0.1216	**0.04123**	8.07285	0.01817	23.785
	Mean	0.1437	0.2024	6.5839	0.19454	**0.06863**	9.372	0.1017	28.10153393
	Median	0.142185	0.20857	7.0242	0.19686	**0.06945**	9.76723	0.04821	27.996
	Worst	0.216855	0.28339	11.057	0.2953	**0.0906**	10.3628	0.2977	32.4397
	Std	0.030715	0.042936	3.08675	0.0503	**0.01411**	1.00302	0.11347	3.13609
F13	Best	9.9177	9.85719	14.169	**5.7551**	6.0079	6.3E+09	17289.9	1.0E+11
	Mean	9.9278	9.9391	105.421	8.9011	**8.705**	8.7E+09	88551.5	1.1E+11
	Median	9.927	9.942	93.656	9.088	**8.9605**	8.8E+09	96054.8	1.1E+11
	Worst	9.9347	9.959	473.7	10.85	**9.391**	11.8E+09	122522	1.15E+11
	Std	**0.0045**	0.0207	102.92	1.2576	0.879	2.03E+09	42982.3	4.36E+09

The best values obtained are in bold.

### Simulation results of optimal power flow

In this section, the detail of the simulation results will be discussed. To authenticate the performance of the LSDO approach, three well-known standard systems were considered as IEEE 30-bus, IEEE 57-bus, and IEEE 118-bus test systems considering two types of renewable energies, which have 24, 33, and 130 control variables, respectively. The main descriptions of these selected grids are tabulated in Table 5. Furthermore, these considered test systems are executed via ten case studies as described in Table 6. The obtained results are compared with the classical version SDO and some state-of-the-art stochastic approaches. The optimal findings are shown in bold text. All the experiment studies are averaged over 30 independent runs, they have been done by using MATLAB R2020a, under Microsoft Windows 10 operating system, and carried out on a personal computer core i5 with 4GB-RAM Processor @1.8GHz. As mentioned before, the power systems under consideration are analyzed through ten distinct case studies, which are defined as follows:

- **Cases 1, 2, 3, 4, 7, 8, 9, and 12:** Without renewable energy resources

These cases represent the primary scenarios focused on reducing fuel costs, power loss, voltage deviation, and emission.

**Table 5:** The basic specifications of the IEEE test systems.

Systems	IEEE-30		IEEE-57		IEEE-118	
Characteristics	Value	Details	Value	Details	Value	Details
Buses	30	^62^	57	^63^	118	^64^
Branches	41	–	80	–	186	–
Generators	6	Buses: 1, 2, 5, 8, 11 and 13	7	Buses: 1, 2, 3, 6, 8, 9 and 12	54	Buses:1, 4, 6, 8, 10, 12, 15, 18, 19, 24, 25, 26, 27, 31, 32, 34, 36, 40, 42, 46, 49, 54, 55, 56, 59, 61, 62, 65, 66, 69, 70, 72, 73, 74, 76, 77, 80, 85, 87, 89, 90, 91, 92, 99, 100, 103, 104, 105, 107, 110, 111, 112, 113 and 116
Slack bus	1	Buses: 1	1	Buses: 1	Buses: 1	69
Wind generators	2	Buses: 5 and 11	2	Buses: 2 and 6	–	–
Solar generators	1	Buses: 13	1	Buses: 9	–	–
Shunts	9	Buses: 10, 12, 15, 17, 20, 21, 23, 24 and 29	3	Buses: 18, 25, and 53	14	Buses:5, 34, 37, 44, 45, 46, 48, 74, 79, 82, 83, 105, 107, 110
Transformers	4	Branches: 11, 12, 15 and 36	17	Buses: 19, 20, 31, 35, 36, 37, 41, 46, 54, 58, 59, 65, 66, 71, 73, 76, and 13	9	Branches:8, 32, 36, 51, 93, 95, 102, 107 and 127
Control variables	24	–	33	–	130	–

**Table 6:** Different case studies.

		*Cost*	*Emission*	$P_{loss}$	*VD*
IEEE-30	Case 1	$\surd$			
	Case 2		$\surd$		
	Case 3			$\surd$	
	Case 4				$\surd$
	Case 5				
	Case 6				
IEEE-57	Case 7	$\surd$			
	Case 8			$\surd$	
	Case 9				$\surd$
	Case 10				
	Case 11				
IEEE-118	Case 12	$\surd$

- **Cases 5, 6, 10 and 11:** With renewable energy resources

These cases are computed based on Eqs. 36 and 37. They are characterized by considering both wind and PV power sources. They depict the core scenario centered on the primary objective of diminishing fuel costs, while accounting for emission, power loss and voltage deviation.

#### IEEE 30-bus test system

The IEEE 30-bus network is the small power system considered in this study. It contains 6 generating units which bus 1 is chosen as the slack bus, 41 branches, 9 shunt reactive power injections, and 4 transformers. The line and bus data are taken from^62^. Additionally, its active and reactive power demands are 283.4*MW* and 126.2*MVAR*, respectively. The voltage limits for all buses are taken between 0.95 and 1.05 p.u. Also, the least as well as greater tap setting for tap changing transformers are 0.9 p.u. and 1.1 p.u., respectively. Moreover, The limits of VAR compensators are assumed to vary between 0 and 5 p.u. The comparison of the obtained results between LSDO and its first version SDO is presented in Table 7. Furthermore, the optimal control variables are displayed in the same tables. As previously illustrated, two scenarios were considered: the first without taking into account renewable energy sources (RESs) whereas the second achieve a reduction in the total fuel cost through the integration of RESs. Specifically, wind power generators have replaced conventional generators at buses 5 and 11, with these wind turbines totaling 25 and 20 units, respectively. Additionally, a PV generator has been introduced to replace the generator at bus 13. The integration and placement of these RESs within the grid are determined based on the methodology outlined in the study by Biswas et al.^51^.

**Figure 7:**
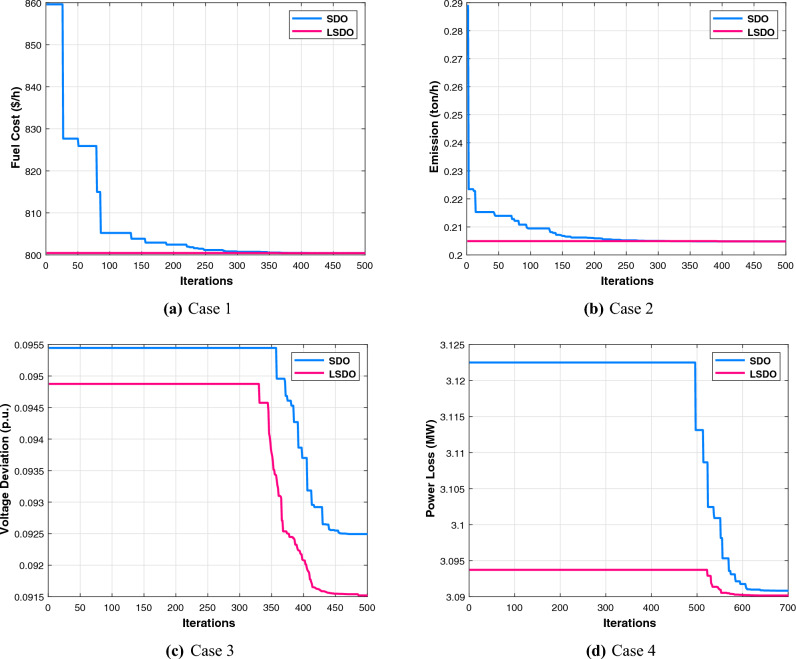
Characteristics of convergence of the proposed LSDO vs SDO for IEEE 30-bus system.

**Figure 8:**
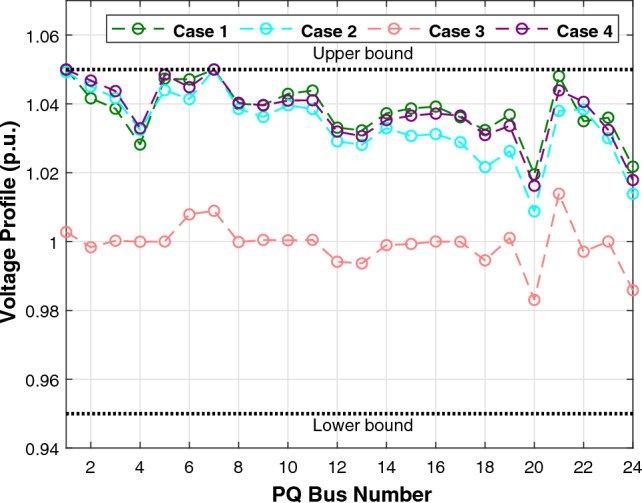
Voltage profiles of PQ buses using the proposed LSDO algorithm for IEEE 30-bus system.

The first case attempted to optimize the quadratic fuel cost. The fitness rates attained are $$800.42 $$/h and $$800.4223 $$/h for LSDO and SDO, respectively. The objective function considering the minimization of total emission is taken as the second case, its best fitness values achieved are 0.20483 ton/h and 0.20484 ton/h. The obtained optimum voltage deviation (VD) for both approaches are 0.09152 p.u. and 0.09249 p.u., respectively. Regarding the power loss minimization, its fitness values recorded 3.0902(*MW*) and 3.0908(*MW*) as demonstrated in the same table. Remarkably, the outcomes reveal that the approach under study produces better solutions compared to its initial version. Additionally, in terms of the convergence characteristics, it can be seen from the evolution curves depicted in Fig. 7 that LSDO converges faster in comparison with SDO. Furthermore, according to the constraints satisfaction, Fig. 8 proves the effectiveness of LSDO-based SF in answering all system constraints. On the other hand, some of the published results are competitive with those generated by the LSDO technique, they offered better solutions as listed in Table 8 However, it can be observed carefully that certain of their voltage load buses are violated. Otherwise, the highest voltage deviation value that must be produced is 1.2*p*.*u*. of all PQ buses. More precisely, the infeasibility solutions footnoted in Table 8 can be explained in the following lines. For case 1 and as reported in^9^, the EWOA voltage deviation value is higher than 1.2*p*.*u*., in which all load buses exceed the maximum bound except buses 26 and 30. Ref.^12^ reports a VD value of 1.9652*p*.*u*. and a violation of all load voltage. The DSA approach^13^ has one bus violation at bus number 3. The optimum result taken from^15^ is an infeasible solution due to the violation in nodes 3, 4, 6, 7, 12, 14, 15, 16, 23, 25, 27, 28, 29, and 30. Referring to^16^, the best results stated for all Rao variants are also infeasible, there are voltage loads violations in buses 3, 4, 10, and 12 for Rao-1, and in buses 3, 4, 6, 10, 12, 14, 15, 16, 17, 20, 21, 22, 23, and 27 for Rao-2, and also in the buses 3, 4, 6, 10, 12, 14, 15, 16, 17, 20, 21, 22, 23, 27, and 29 for Rao-3. Additionally, the minimum fitness value reported in MSA Ref.^10^ for the emission case is too an infeasible solution owing to nodes 3 and 12.

**Table 7:** The obtained results of the proposed LSDO as well as the original SDO techniques for IEEE 30-bus.

Control variables	Min	Max	Case 1		Case 2		Case 3		Case 4	
			SDO	LSDO	SDO	LSDO	SDO	LSDO	SDO	LSDO
P$${}_{g2}$$	20	80	48.8654	48.6153	67.7502	67.5837	29.7910	73.8103	79.9826	79.9790
P$${}_{g5}$$	15	50	21.4049	21.4015	49.9997	49.9973	24.7736	30.3605	49.9932	49.9976
P$${}_{g8}$$	10	35	21.1015	21.3178	34.9994	34.9986	28.3303	15.7616	34.9998	34.9996
P$${}_{g11}$$	10	30	11.9564	11.6592	29.9974	29.9934	22.9054	19.1346	29.9951	29.9906
P$${}_{g13}$$	12	40	12.0049	12.0101	40.0000	39.9999	16.7438	20.7435	39.9938	39.9586
V$${}_{g1}$$	0.95	1.1	1.0823	1.0833	1.0642	1.0613	1.0229	1.0194	1.0612	1.0607
V$${}_{g2}$$	0.95	1.1	1.0628	1.0636	1.0598	1.0548	1.0115	1.0131	1.0572	1.0567
V$${}_{g5}$$	0.95	1.1	1.0322	1.0316	1.0377	1.0309	1.0189	1.0187	1.0363	1.0359
V$${}_{g8}$$	0.95	1.1	1.0373	1.0371	1.0398	1.0388	1.0046	1.0042	1.0450	1.0435
V$${}_{g11}$$	0.95	1.1	1.0787	1.0867	1.0827	1.0855	1.0281	1.0319	1.0888	1.0821
V$${}_{g13}$$	0.95	1.1	1.0485	1.0469	1.0564	1.0595	1.0019	1.0030	1.0477	1.0544
Q$${}_{c10}$$	0	5	3.8509	3.6239	1.8352	2.1188	4.6393	4.7964	0.0422	1.8110
Q$${}_{c12}$$	0	5	2.5683	0.8028	1.0072	2.6961	1.0379	1.2061	3.6747	3.0673
Q$${}_{c15}$$	0	5	2.9863	4.0522	3.1281	3.2531	4.8235	4.9231	3.2459	4.3718
Q$${}_{c17}$$	0	5	4.7312	4.9483	4.1349	2.8464	0.5307	0.1258	4.7300	3.6981
Q$${}_{c20}$$	0	5	4.1167	3.7935	4.3456	2.8350	4.9772	4.9837	4.2526	3.5623
Q$${}_{c21}$$	0	5	4.9293	4.9778	2.1946	4.3615	4.9418	4.9478	4.9824	4.9991
Q$${}_{c23}$$	0	5	3.0081	2.6271	2.1445	3.4747	4.8950	4.9863	3.9797	3.6530
Q$${}_{c24}$$	0	5	4.9895	4.8870	2.0953	3.0982	4.9451	4.9916	4.9381	4.9868
Q$${}_{c29}$$	0	5	2.3247	2.3386	3.7650	2.3354	2.3090	2.0533	3.2013	2.5615
T$${}_{11}$$	0.9	1.1	1.0291	1.0281	1.0284	1.0229	1.0439	1.0469	1.0592	1.0187
T$${}_{12}$$	0.9	1.1	0.9457	0.9534	0.9419	0.9280	0.9001	0.9014	0.9200	0.9610
T$${}_{15}$$	0.9	1.1	0.9734	0.9681	0.9828	0.9989	0.9612	0.9659	0.9855	0.9951
T$${}_{36}$$	0.9	1.1	0.9730	0.9724	0.9850	0.9690	0.9645	0.9619	0.9825	0.9798
Fuel Cost ($/h)	–	–	800.4223	**800.42**	944.7892	944.4218	816.3608	832.8700	967.5433	967.4535
Emission (t/h)	–	–	0.36609	0.36708	0.20484	**0.20483**	0.34210	0.27505	0.20726	0.20727
VD (p.u.)	–	–	0.9075	0.91576	0.81458	0.89701	0.09249	**0.09152**	0.91790	0.90258
$$P_{L}$$ (MW)	–	–	9.006	9.0202	3.2821	3.2522	8.9451	7.8115	3.0908	**3.0902**
P$${}_{g1}$$	50	200	177.0728	177.4163	63.9355	64.0793	169.80105	131.40115	51.52628	51.56473

The best values obtained are in bold.

**Table 8:** Comparison of the LSDO and SDO algorithms and previous studies for IEEE 30-bus system.

Approaches	IEEE 30-bus	IEEE 57-bus	IEEE 118-bus
Case 1: fuel cost ($/h)			
LSDO	**800.42**	**41667.719**	**137105.9933**
SDO	800.4223	41668.7587	139923.6969
ACO^8^	802.097	–	138809.3896
EWOA^9^	$$799.210^{*}$$	–	142756.67
MSA^10^	800.5099	41673.72	–
AGSO^11^	801.75	–	–
ICBO^12^	$$799.0353^{*}$$	41697.33	–
DSA^13^	$$800.3887^{*}$$	41686.82	–
IWO^14^	800.92	41768.49	–
ISA^15^	$$799.2776^{*}$$	41676.9466	–
Rao-1^16^	800.4391	41771.1088	$$129241.1787^{*}$$
Rao-2^16^	$$799.9918^{*}$$	41872.0668	$$129256.5242^{*}$$
Rao-3^16^	$$799.9683^{*}$$	$$41659.2621^{*}$$	$$129220.6794^{*}$$
SSA^17^	–	41672.30	–
DE^65^	–	41682	–
HHODE^66^	800.9959	–	–
SKH^18^	800.5141	–	–
SOS^19^	801.5733	–	–
HHO^67^	801.8290	–	–
PSO/APO^68^	801.708	–	–
Case 2: Emission (ton/h)			
LSDO	**0.20483**	–	–
SDO	0.20484	–	–
MSA^10^	$$0.20482^{*}$$	–	–
AGSO^11^	0.2059	–	–
DSA^13^	0.20583	–	–
IWO^14^	0.20521	–	–
HHO^67^	0.2850	–	–
Case 3: VD (p.u.)			
LSDO	**0.091521**	**0.62165**	–
SDO	0.092494	0.63354	–
Rao-1^16^	0.1031	0.9882	–
Rao-2^16^	0.0993	0.7645	–
Rao-3^16^	0.1001	$$0.5725^{*}$$	–
SSA^17^	–	0.7569	–
DE^65^	–	$$0.5839^{*}$$	–
TSA^58^	–	0.72	–
Case 4: Power Loss (MW)			
LSDO	**3.0902**	**10.2332**	–
SDO	3.0908	10.4552	–
MSA^10^	3.1005	–	–
DSA^13^	3.0945	–	–
HHO^67^	3.49	–	–
IWO^14^	4.847	24.19	–
DE^65^	–	10.2642	–
SSA^17^	–	11.32	–
TSA^58^	–	12.473	–

$$*$$ : Infeasible solution.

**Figure 9:**
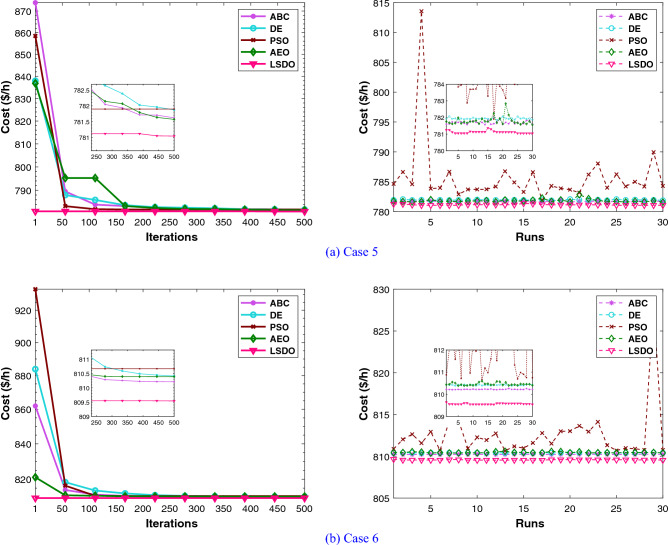
Comparison of convergence and run of LSDO vs state-of-the-art algorithms for cases 5 and 6.

**Figure 10:**
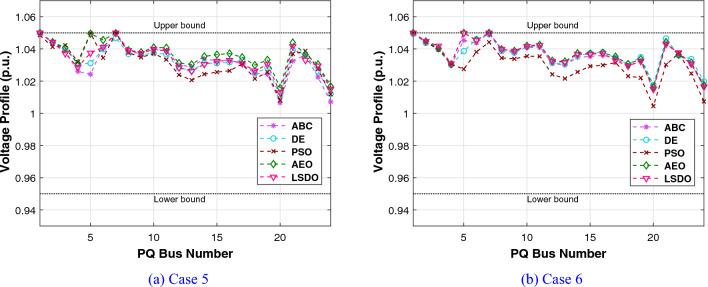
Voltage profile of PQ buses for IEEE-30 REs cases.

Regarding the RESs scenario, the results obtained from the proposed LSDO approach are compared with those from the SDO method, as well as four re-implemented techniques, namely: artificial ecosystem optimization (AEO)^69^, particle swarm optimization (PSO)^60^, artificial bee colony (ABC)^61^, and deferential evolution (DE)^59^. Simulation results were generated using 50 populations, and their convergence was assessed by analyzing the plots obtained from each case over 500 iterations. To ensure statistical reliability, a total of 30 independent runs were conducted for each scenario. The comparative analysis of numerical outcomes across 30 runs for all competing methods is provided in Tables 9, 10. These tables encompass the optimal configurations of control variables, their allowable ranges, and the corresponding numerical best outcomes for each objective. As observed in these presented tables, the LSDO approach showcases a commendable ability to produce competitive results in comparison to both its initial version and other contemporary techniques across case studies. Figure 9 displays the convergence characteristics and distribution runs obtained for each case study of LSDO and the competitor algorithms. This figure illustrates the performance and behavior of the algorithms during the optimization process for the respective scenarios. The convergence curves clearly demonstrate that the LSDO algorithm outperforms its competitors by converging more rapidly towards the optimal solution. This ability to converge faster highlights the efficiency and effectiveness of the LSDO approach in finding high-quality solutions within a shorter number of iterations compared to other competing methods. Furthermore, the obtained optimal PQ voltage profile is depicted in Fig. 10. These visualizations demonstrate that all voltage profile constraints are satisfied, affirming that the feasibility is rigorously examined without any violations of constraints.

**Table 9:** Achieved solutions of the proposed LSDO and its competitors for case 5.

Variables	Min	Max	LSDO	SDO	ABC	AEO	DE	PSO
$$P_{g2}$$	20	80	27.4952	28.1190	29.2181	27.8672	29.3493	27.4586
$$P_{ws1}$$	0	75	42.5964	42.4655	43.6871	43.3611	44.0153	44.4882
$$P_{g8}$$	10	35	10.0003	10.0168	10.0000	10.0000	10.0070	10.0000
$$P_{ws2}$$	0	60	36.3138	35.8283	37.2811	37.2411	36.9425	36.9460
$$P_{ss}$$	0	50	37.6781	37.6680	33.8187	35.5632	33.7036	35.1731
$$V_{g1}$$	0.95	1.1	1.0712	1.0736	1.0705	1.0715	1.0720	1.0764
$$V_{g2}$$	0.95	1.1	1.0567	1.0566	1.0547	1.0569	1.0570	1.0610
$$V_{g5}$$	0.95	1.1	1.0350	1.0318	1.0266	1.0350	1.0347	1.0356
$$V_{g8}$$	0.95	1.1	1.0339	1.0372	1.0386	1.0381	1.0383	1.0399
$$V_{g11}$$	0.95	1.1	1.0576	1.0861	1.0377	1.0969	1.0679	1.1000
$$V_{g13}$$	0.95	1.1	1.0640	1.0466	1.0582	1.0532	1.0476	1.0301
$$Q_{c10}$$	0	5	0.9424	4.1096	5.0000	1.6484	3.6814	2.6581
$$Q_{c12}$$	0	5	2.7784	4.6240	4.0577	0.9597	0.9146	5.0000
$$Q_{c15}$$	0	5	4.5231	4.2841	4.0942	2.6031	4.7725	1.8142
$$Q_{c17}$$	0	5	4.7475	2.1458	5.0000	2.5202	4.2278	5.0000
$$Q_{c20}$$	0	5	2.5055	2.5539	4.7588	3.6337	3.7487	1.9695
$$Q_{c21}$$	0	5	4.3127	4.5084	5.0000	3.8520	4.5469	3.5004
$$Q_{c23}$$	0	5	1.6562	2.2216	2.1084	3.3026	4.3059	4.4025
$$Q_{c24}$$	0	5	4.9079	4.1379	4.9920	4.6778	3.6728	4.1748
$$Q_{c29}$$	0	5	2.3821	1.9546	3.1337	1.8963	2.0644	3.2827
$$T_{11}$$	0.9	1.1	1.0224	1.0453	1.0571	1.0272	1.0595	1.0093
$$T_{12}$$	0.9	1.1	0.9262	0.9400	0.9000	0.9470	0.9245	1.0445
$$T_{15}$$	0.9	1.1	1.0018	0.9784	0.9976	0.9811	0.9814	0.9397
$$T_{36}$$	0.9	1.1	0.9718	0.9751	0.9903	0.9720	0.9767	0.9846
$$T_C$$ ($/h)	–	–	**781.0465**	781.0633	781.5679	781.5613	781.8429	781.8984
$$W_C$$ ($/h)	–	–	240.1276	238.0496	247.1789	245.9129	247.1596	248.8258
$$S_C$$ ($/h)	–	–	103.6752	103.6431	91.2099	97.1776	91.1570	95.9508
*E* (t/h)	–	–	1.7623	1.7622	1.7683	1.7622	1.7648	1.7623
*VD* (p.u.)	–	–	0.8136	0.8011	0.7702	0.8816	0.7889	0.7628
$$P_{L}$$ (MW)	–	–	5.5916	5.6057	5.5719	5.5407	5.5521	5.5739
P$${}_{g1}$$	50	140	134.9079	134.9080	134.9668	134.9079	134.9343	134.9079

The best values obtained are in bold.

**Table 10:** Achieved solutions of the proposed LSDO and its competitors for case 6.

Variables	Min	Max	LSDO	SDO	ABC	AEO	DE	PSO
$$P_{g2}$$	20	80	32.8384	32.8729	31.9959	33.6714	34.0393	33.7709
$$P_{ws1}$$	0	75	45.6214	45.8477	45.3124	46.3784	46.0525	46.5292
$$P_{g8}$$	10	35	10.0018	10.0038	10.0049	10.0000	10.0071	10.0000
$$P_{ws2}$$	0	60	38.8516	38.5311	38.2687	39.0636	39.2151	39.2387
$$P_{ss}$$	0	50	37.6834	37.6979	39.8551	35.5634	35.2538	35.1733
$$V_{g1}$$	0.95	1.1	1.0706	1.0691	1.0713	1.0696	1.0710	1.0708
$$V_{g2}$$	0.95	1.1	1.0559	1.0560	1.0559	1.0563	1.0565	1.0570
$$V_{g5}$$	0.95	1.1	1.0345	1.0335	1.0334	1.0349	1.0345	1.0359
$$V_{g8}$$	0.95	1.1	1.0395	1.0383	1.0374	1.0376	1.0381	1.0367
$$V_{g11}$$	0.95	1.1	1.0829	1.0949	1.0805	1.0752	1.0781	1.0607
$$V_{g13}$$	0.95	1.1	1.0492	1.0519	1.0473	1.0546	1.0518	1.0446
$$Q_{c10}$$	0	5	0.3339	4.0594	3.9513	4.3021	2.1917	1.3419
$$Q_{c12}$$	0	5	2.0384	1.1285	4.2833	2.4768	1.0254	5.0000
$$Q_{c15}$$	0	5	4.2634	4.2578	3.4435	3.1776	2.8819	5.0000
$$Q_{c17}$$	0	5	4.9328	4.6238	4.7729	4.9975	3.8599	5.0000
$$Q_{c20}$$	0	5	3.7234	4.5632	3.9906	4.8392	3.7065	1.3802
$$Q_{c21}$$	0	5	4.9655	4.9980	4.9840	4.9269	4.8417	4.1186
$$Q_{c23}$$	0	5	4.1069	1.5115	2.9477	2.9978	2.9213	5.0000
$$Q_{c24}$$	0	5	4.6444	4.9451	5.0000	4.9772	4.4943	5.0000
$$Q_{c29}$$	0	5	1.8727	2.3339	2.1057	2.1668	2.1261	4.6306
$$T_{11}$$	0.9	1.1	1.0381	1.0240	1.0229	1.0027	1.0510	1.0625
$$T_{12}$$	0.9	1.1	0.9365	0.9803	0.9695	0.9862	0.9104	0.9000
$$T_{15}$$	0.9	1.1	0.9804	0.9817	0.9802	0.9894	0.9817	0.9947
$$T_{36}$$	0.9	1.1	0.9809	0.9755	0.9724	0.9728	0.9688	0.9993
$$C_T$$ ($/h)	–	–	**809.5379**	809.5406	810.2063	810.3869	810.3891	810.6643
$$C_{W}$$ ($/h)	–	–	259.4633	259.1410	256.3126	262.9280	262.2946	264.0941
$$C_{S}$$ ($/h)	–	–	103.6924	103.7418	111.8707	97.1782	96.0590	95.9513
E (t/h)	–	–	0.8875	0.8898	0.8652	0.9034	0.9104	0.9033
VD (p.u.)	–	–	0.8509	0.8870	0.8691	0.8965	0.8831	0.7246
$$P_{L}$$ (MW)	–	–	5.0458	5.0472	5.0390	5.0358	5.0618	5.0694
P$${}_{g1}$$	50	140	123.4492	123.4939	123.0019	123.7589	123.8941	123.7573

The best values obtained are in bold.

#### IEEE 57-bus test system

**Figure 11:**
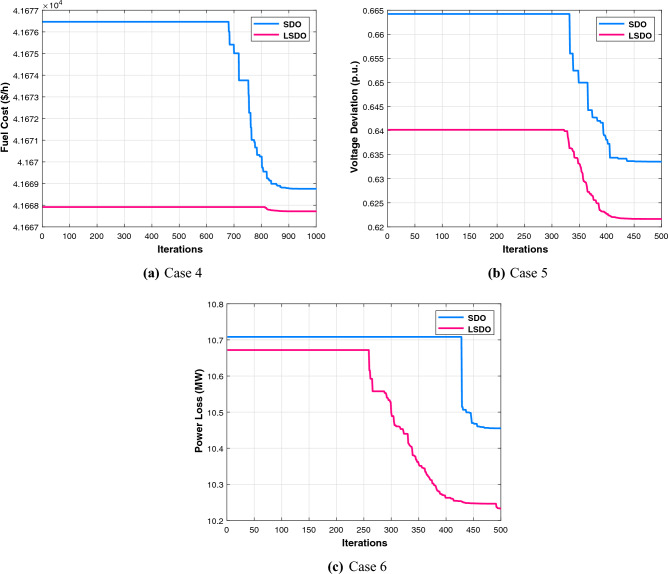
Characteristics of convergence of the proposed LSDO vs SDO for IEEE 57-bus system.

**Figure 12:**
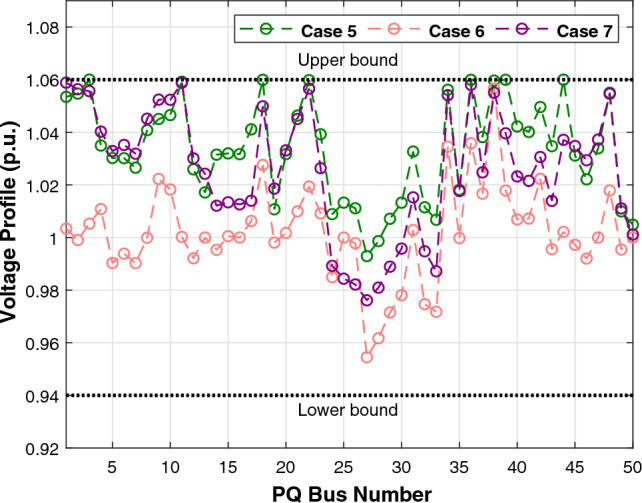
Voltage profiles of PQ buses using the proposed LSDO for IEEE 57-bus system.

To check the scalability of the algorithm under study, the medium IEEE 57-bus test system is examined. This network contains seven generators and the slack generator is at bus 1, 80 branches, 50 load buses, three shunt reactive power injections, and 15 transformers. Its active and reactive power demands are 1250.8 MW and 336.4 MVAR, respectively. This system has total of 33 control variables for the OPF problem, their bounds and the achieved optimized values for the three objective functions are listed in Table 11. The obtained fitness values from the LSDO and SDO algorithms for fuel cost, voltage deviation, and power loss are ($$41667.7190 $$/h–$$41668.7587 $$/h), (0.62165–0.63354 p.u.), and (10.2332–10.4552 MW), respectively. Based on these outcomes, it is obvious that the modified approach LSDO provides the optimum fitness value of all objective functions as compared to its classical version SDO. Figure 11 illustrates the convergence of the functions evolution of both algorithms. Based on these curves, it is clearly seen that the LSDO has a steady and speed convergence acceleration toward the global optimum than SDO. Table 8 gives a comparative study with other stochastic approaches stated in the literature, the listed best value for this system stated in^11^ case 1, is an infeasible solution, it knows a load voltage violations at buses 7, 18, 25, 29, 41, and 45. For the voltage deviation case, the Rao-3^16^ seems to converge to the optimum solution than the suggested LSDO optimizer. However, after careful observation, node 25 violates the constraints with voltage values of 1.06687*p*.*u*.. In addition, the optimum value of DE algorithm^65^ is due to higher limits for the shunt compensators. Nevertheless, the optimizer under study LSDO gives a better solution without any constraint violation, as shown in Fig. 12 The voltage of the 50 loads buses (PQ bus) are satisfying all system constraints for all cases, which proves that all system constraints are checked.

**Table 11:** The obtained results of the proposed LSDO as well as the original SDO techniques for IEEE 57-bus.

Control variables	Min	Max	Case 1		Case 2		Case 3	
			SDO	LSDO	SDO	LSDO	SDO	LSDO
P$${}_{g2}$$	30	100	90.6263	90.1633	77.1444	76.0949	55.3909	30.0065
P$${}_{g3}$$	40	140	45.4041	44.7779	54.7384	63.8062	123.6990	112.3814
P$${}_{g6}$$	30	100	70.3122	74.0789	63.0807	54.1637	91.3919	91.6064
P$${}_{g8}$$	100	550	460.9184	460.5164	315.6719	405.2965	331.1286	326.6915
P$${}_{g9}$$	30	100	93.5818	93.4810	48.4015	68.8195	99.1253	99.7742
P$${}_{g12}$$	100	410	361.7702	360.5197	263.3974	261.0801	409.6722	409.3781
V$${}_{g1}$$	0.95	1.1	1.0629	1.0616	1.0234	1.0316	1.0598	1.0656
V$${}_{g2}$$	0.95	1.1	1.0609	1.0600	1.0169	1.0211	1.0584	1.0611
V$${}_{g3}$$	0.95	1.1	1.0540	1.0544	1.0104	1.0082	1.0621	1.0612
V$${}_{g5}$$	0.95	1.1	1.0620	1.0604	1.0061	1.0022	1.0627	1.0600
V$${}_{g8}$$	0.95	1.1	1.0724	1.0741	1.0241	1.0155	1.0693	1.0630
V$${}_{g9}$$	0.95	1.1	1.0460	1.0465	1.0048	1.0010	1.0462	1.0451
V$${}_{g12}$$	0.95	1.1	1.0466	1.0458	1.0268	1.0302	1.0491	1.0544
Q$${}_{c18}$$	0	20	10.2617	12.8767	10.2150	5.6659	5.1816	10.8859
Q$${}_{c25}$$	0	20	13.3938	13.3632	12.9599	18.4242	8.6958	13.9401
Q$${}_{c53}$$	0	20	11.7915	10.7761	19.5538	17.5290	8.9473	15.8698
T$${}_{19}$$	0.9	1.1	1.0126	1.0880	1.0387	0.9875	1.0004	1.0319
T$${}_{20}$$	0.9	1.1	0.9896	0.9384	0.9795	1.0001	0.9736	0.9777
T$${}_{31}$$	0.9	1.1	1.0193	1.0140	0.9709	0.9686	1.0101	0.9823
T$${}_{35}$$	0.9	1.1	1.0502	1.0151	1.0337	1.0352	0.9789	1.0055
T$${}_{36}$$	0.9	1.1	0.9683	1.0128	1.0041	1.0575	1.0327	0.9792
T$${}_{37}$$	0.9	1.1	1.0208	1.0284	1.0281	1.0049	1.0469	0.9943
T$${}_{41}$$	0.9	1.1	0.9965	0.9961	0.9811	0.9869	1.0084	0.9969
T$${}_{46}$$	0.9	1.1	0.9607	0.9572	0.9179	0.9242	0.9937	0.9780
T$${}_{54}$$	0.9	1.1	0.9526	0.9179	0.9010	0.9027	0.9548	0.9255
T$${}_{58}$$	0.9	1.1	0.9780	0.9785	0.9291	0.9343	0.9799	0.9822
T$${}_{59}$$	0.9	1.1	0.9633	0.9591	0.9740	0.9702	0.9671	0.9845
T$${}_{65}$$	0.9	1.1	0.9721	0.9713	1.0055	1.0061	0.9730	0.9979
T$${}_{66}$$	0.9	1.1	0.9339	0.9341	0.9030	0.9011	0.9480	0.9550
T$${}_{71}$$	0.9	1.1	0.9630	0.9687	0.9636	0.9537	1.0103	0.9729
T$${}_{73}$$	0.9	1.1	1.0165	0.9973	0.9622	0.9987	1.0563	0.9501
T$${}_{76}$$	0.9	1.1	0.9867	0.9685	0.9269	0.9020	1.0049	0.9802
T$${}_{80}$$	0.9	1.1	0.9881	0.9863	0.9994	0.9793	1.0059	0.9879
Fuel Cost ($/h)	–	–	41668.7587	**41667.7190**	50990.2155	45681.7670	43796.8998	43612.0992
Emission (ton/h)	–	–	1.35777	1.35367	1.78130	1.49988	1.10023	1.15161
Voltage Deviation (p.u.)	–	–	1.61981	1.69704	0.63354	**0.62165**	1.44485	1.53517
Power Loss (MW)	–	–	14.8667	14.9243	29.7593	23.1825	10.4552	**10.2332**
P$${}_{g1}$$	0	576	143.05363	142.18709	458.12503	344.72168	150.84732	191.19500

The best values obtained are in bold.

**Table 12:** Achieved solutions of the proposed LSDO and its competitors for case 10.

Variables	Min	Max	LSDO	SDO	ABC	AEO	DE	PSO
$$P_{ws1}$$	0	150	149.9968	149.9149	150.0000	149.9607	144.5646	150.0000
$$P_{g3}$$	40	140	40.0596	44.6584	132.8028	41.2343	68.4754	40.0000
$$P_{ws2}$$	0	150	149.9959	149.9181	150.0000	149.9662	149.6426	150.0000
$$P_{g8}$$	100	550	378.8350	384.6096	223.1031	387.2554	402.3747	390.3843
$$P_{ss}$$	0	120	119.9969	119.9445	105.4476	119.9906	107.8691	120.0000
$$P_{g12}$$	100	410	315.6892	309.6422	316.7675	306.9151	292.5309	306.6320
$$V_{g1}$$	0.95	1.1	1.0596	1.0336	1.0388	1.0402	1.0431	1.0585
$$V_{g2}$$	0.95	1.1	1.0625	1.0301	1.0336	1.0378	1.0314	1.0534
$$V_{g3}$$	0.95	1.1	1.0572	1.0249	1.0227	1.0344	1.0375	1.0531
$$P_{g6}$$	0.95	1.1	1.0653	1.0399	1.0327	1.0572	1.0369	1.0590
$$V_{g8}$$	0.95	1.1	1.0449	1.0447	1.0465	1.0583	1.0316	1.0409
$$V_{g9}$$	0.95	1.1	1.0297	1.0229	1.0169	1.0380	1.0211	1.0287
$$V_{g12}$$	0.95	1.1	1.0372	1.0271	1.0168	1.0501	1.0421	1.0566
$$Q_{c18}$$	0	20	18.7913	14.6511	3.9084	8.7065	10.3670	16.8329
$$Q_{c25}$$	0	20	16.8467	15.7788	10.4987	13.8436	13.0369	19.9816
$$Q_{c53}$$	0	20	5.5652	7.2514	13.8125	12.7174	19.0196	9.9873
$$T_{19}$$	0.9	1.1	1.0668	0.9850	0.9970	1.0219	0.9398	1.0562
$$T_{20}$$	0.9	1.1	0.9928	1.0229	1.0194	1.0236	1.0274	1.0978
$$T_{31}$$	0.9	1.1	1.0072	1.0090	1.0094	1.0337	0.9462	0.9600
$$T_{35}$$	0.9	1.1	1.0818	1.0780	1.0747	1.0236	0.9998	1.0755
$$T_{36}$$	0.9	1.1	0.9311	0.9868	0.9687	0.9751	1.0441	0.9990
$$T_{37}$$	0.9	1.1	1.0398	1.0483	1.1000	1.0329	1.0677	1.0646
$$T_{41}$$	0.9	1.1	1.0079	0.9762	0.9731	1.0094	0.9680	0.9766
$$T_{46}$$	0.9	1.1	0.9644	0.9895	0.9000	0.9327	0.9500	0.9952
$$T_{54}$$	0.9	1.1	1.0226	0.9595	0.9703	0.9961	0.9332	0.9955
$$T_{58}$$	0.9	1.1	0.9813	0.9512	0.9433	0.9578	0.9568	1.0399
$$T_{59}$$	0.9	1.1	0.9922	0.9491	0.9541	0.9801	1.0382	1.0591
$$T_{65}$$	0.9	1.1	0.9899	1.0342	1.0295	1.0012	0.9597	1.0172
$$T_{66}$$	0.9	1.1	0.9448	0.9519	0.9415	0.9786	0.9355	0.9062
$$T_{71}$$	0.9	1.1	0.9827	0.9549	0.9303	0.9961	0.9840	1.0383
$$T_{73}$$	0.9	1.1	0.9919	1.0501	0.9551	1.0316	0.9147	0.9726
$$T_{76}$$	0.9	1.1	0.9653	1.0026	1.1000	0.9617	1.0116	0.9549
$$T_{80}$$	0.9	1.1	1.0546	1.0213	0.9862	0.9890	1.0895	0.9746
$$C_T$$ ($/h)	–	–	**26491.8072**	26529.4584	30539.0423	26498.1112	27458.7947	26565.5122
$$C_{W}$$ ($/h)	–	–	1231.2046	1230.4574	1231.2389	1230.8971	1204.5255	1231.2389
$$C_{S}$$ ($/h)	–	–	461.9679	461.6379	395.9494	463.0663	407.6080	462.7849
E (t/h)	–	–	0.8984	0.9016	0.6918	0.9082	0.9253	0.9177
VD (p.u.)	–	–	1.2561	1.0096	1.1084	1.1498	1.2434	1.5757
$$P_{L}$$ (MW)	–	–	17.1841	18.0821	18.6802	17.9220	21.1848	19.9461
P$${}_{g1}$$	0	575.88	113.4107	110.1944	191.3593	113.3998	106.5275	113.7299

The best values obtained are in bold.

**Figure 13:**
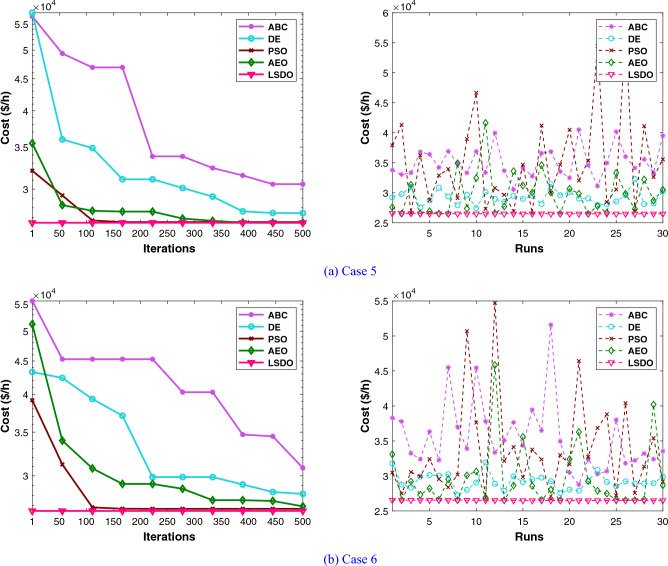
Comparison of convergence and run of LSDO vs state-of-the-art algorithms for cases 5 and 6.

**Figure 14:**
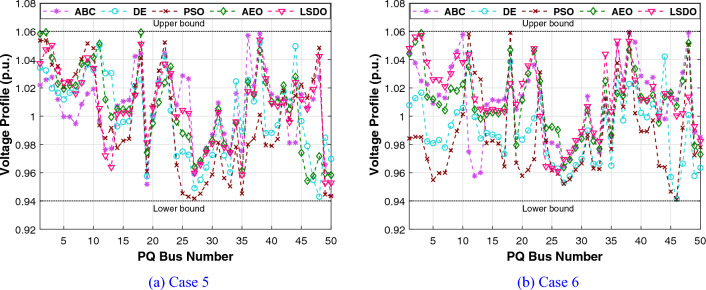
Voltage profile of PQ buses for IEEE-57 REs cases.

In the context of the renewable energy sources (RESs), the outcomes achieved using the proposed LSDO approach are compared with those obtained from the SDO method, AEO^69^, PSO^60^, ABC^61^, and DE^59^ approaches. This comparison assesses the performance and efficacy of the LSDO approach in optimizing the RESs integration and addressing the associated objectives. The comparative analysis of numerical outcomes from the 30 independent runs for all competing methods is provided in Tables 12, 13. These tables present the optimal configurations of allowable ranges, control variables, and the corresponding best numerical outcomes achieved for each objective. The data in these tables offer valuable insights into the performance and effectiveness of each method in solving the optimization problems in the given scenario. Figure 13 presents the convergence characteristics and distribution runs obtained for each case study of the LSDO approach and the competitor algorithms. This figure indicates that the LSDO algorithm outperforms its competitors by converging more rapidly towards the optimal solution. The figure emphasizes the robustness and competitiveness of the LSDO approach in addressing the optimization challenges in this considered system. Additionally, the obtained optimal PQ voltage profile is illustrated in Fig. 14. These visualizations effectively demonstrate that all voltage profile constraints are met, thereby confirming that the feasibility of the solutions is thoroughly verified without any violations of constraints. The optimal PQ voltage profile adheres to the operational limits, ensuring a stable and reliable performance of the power system. These qualitative and quantitative results illustrate that the LSDO approach exhibits a commendable capability to generate competitive solutions, performing favorably in comparison to both its initial version and other contemporary techniques across the different IEEE-57 case studies.

**Table 13:** Achieved solutions of the proposed LSDO and its competitors for case 11.

Variables	Min	Max	LSDO	SDO	ABC	AEO	DE	PSO
$$P_{g2}$$	0	150	149.9869	149.9979	101.6808	149.9480	149.9373	150.0000
$$P_{g3}$$	40	140	40.0190	41.4222	75.5268	41.9813	65.0723	40.0000
$$P_{g6}$$	0	150	149.9852	149.9998	150.0000	149.9726	143.1387	149.5369
$$P_{g8}$$	100	550	384.0763	370.1775	397.0061	392.0774	371.4336	367.3130
$$P_{g9}$$	0	120	119.9840	119.9928	114.9231	119.9816	114.5233	120.0000
$$P_{g12}$$	100	410	309.2269	323.9497	303.1151	305.3395	285.2056	324.1490
$$V_{g1}$$	0.95	1.1	1.0468	1.0458	1.0844	1.0421	1.0247	1.0294
$$V_{g2}$$	0.95	1.1	1.0480	1.0402	1.0736	1.0441	1.0227	1.0232
$$V_{g3}$$	0.95	1.1	1.0435	1.0381	1.0505	1.0392	1.0048	0.9904
$$P_{g6}$$	0.95	1.1	1.0653	1.0473	1.0397	1.0622	1.0206	0.9918
$$V_{g8}$$	0.95	1.1	1.0651	1.0314	1.0225	1.0731	1.0265	0.9865
$$V_{g9}$$	0.95	1.1	1.0436	1.0121	1.0135	1.0331	0.9951	0.9650
$$V_{g12}$$	0.95	1.1	1.0498	1.0293	1.0382	1.0166	1.0033	0.9821
$$Q_{c18}$$	0	20	15.7069	4.7349	13.6560	16.3110	17.1528	0.0000
$$Q_{c25}$$	0	20	13.8184	14.6576	16.4312	8.7039	16.8865	20.0000
$$Q_{c53}$$	0	20	12.5339	13.4582	12.6849	8.2655	0.9974	0.0000
$$T_{19}$$	0.9	1.1	1.0664	1.0617	1.0539	1.0525	0.9865	0.9155
$$T_{20}$$	0.9	1.1	0.9797	1.0693	1.0982	0.9991	0.9696	0.9029
$$T_{31}$$	0.9	1.1	0.9923	1.0077	1.0287	0.9962	1.0061	0.9438
$$T_{35}$$	0.9	1.1	0.9882	0.9602	0.9987	0.9187	0.9579	1.0336
$$T_{36}$$	0.9	1.1	1.0285	0.9671	1.0419	1.0095	1.0001	0.9000
$$T_{37}$$	0.9	1.1	0.9924	0.9521	1.0031	1.0264	0.9825	0.9908
$$T_{41}$$	0.9	1.1	1.0048	0.9921	0.9903	1.0054	1.0146	1.0186
$$T_{46}$$	0.9	1.1	0.9856	0.9922	0.9632	0.9494	0.9748	0.9906
$$T_{54}$$	0.9	1.1	0.9241	1.0152	1.0358	0.9083	0.9499	0.9292
$$T_{58}$$	0.9	1.1	0.9668	1.0236	0.9655	0.9638	0.9664	0.9000
$$T_{59}$$	0.9	1.1	0.9893	0.9714	0.9479	0.9600	0.9439	0.9529
$$T_{65}$$	0.9	1.1	1.0178	0.9550	1.0245	0.9954	0.9331	1.0066
$$T_{66}$$	0.9	1.1	0.9478	0.9059	0.9299	0.9476	0.9318	0.9000
$$T_{71}$$	0.9	1.1	0.9689	0.9416	0.9453	0.9968	0.9411	0.9100
$$T_{73}$$	0.9	1.1	0.9822	1.0071	0.9218	0.9889	1.0581	1.0111
$$T_{76}$$	0.9	1.1	0.9903	0.9966	0.9575	0.9753	0.9156	0.9003
$$T_{80}$$	0.9	1.1	1.0264	0.9753	0.9487	0.9738	0.9815	0.9000
$$C_T$$ ($/h)	–	–	**26490.4174**	26560.9184	28755.3530	26500.9984	27247.6331	26695.5204
$$C_{W}$$ ($/h)	–	–	1231.1081	1231.2280	1009.1192	1230.8696	1198.2998	1229.0353
$$C_{S}$$ ($/h)	–	–	461.2707	461.3112	439.2436	461.9111	437.0527	463.1334
E (t/h)	–	–	0.9039	0.8874	0.9483	0.9164	0.8554	0.8870
VD (p.u.)	–	–	1.1424	1.3345	1.2478	1.0188	1.0682	1.4830
$$P_{L}$$ (MW)	–	–	17.1879	17.6400	19.6197	17.6620	19.5208	20.5897
P$${}_{g1}$$	0	575.88	114.7097	112.9001	128.1678	109.1615	141.0100	120.3908

The best values obtained are in bold.

#### IEEE 118-bus test system

**Figure 15:**
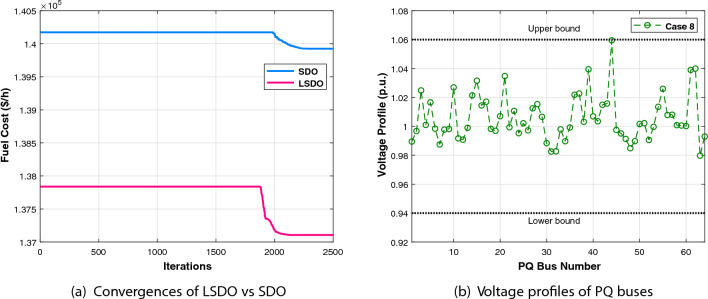
Convergences and voltage profiles of PQ buses for IEEE 118-bus system.

In this part, The LSDO approach has been demonstrated on the IEEE 118-bus test system as a large scale problem in order to affirm the robustness of this suggested technique. The system active and reactive power demands are 4242 MW and 1439 MVAr, respectively. This network contains 118 nods, 54 generators in which the slack generator is at node 69, 186 branches, 14 shunt elements, 9 transformers tap, and 130 control variables. Voltage, shunt capacitors, and transformers tap limits are considered in the range of [0.95–1.1 p.u.], [0–25 p.u.] p.u., and [0.9–1.1 p.u.], respectively. Table 14 outlines the optimal values of the objective functions and their optimal control variables for both SDO and its LSDO variant. The total generation fuel cost for both LSDO and SDO are $$137105.9933 $$ /h and $$139923.6969 $$ /h, respectively. From these results, we note a decrease in the objective function for the improved approach. Besides, according to comparison results described in Table 8, it is apparent that the proposed approach gives a better solution compared to the other meta-heuristic algorithms stated in Some of the recent literature. Moreover, in the field of convergence characteristics, the graphical comparisons between SDO and LSDO of the fuel cost function are illustrated in Fig. 15a. The convergence and rapid speed are marked for the enhanced method LSDO, in which it converges more steadily toward the optimum solution. Similar to the aforementioned systems, all constraints are diligently satisfied using the superiority of feasible solution SF constraint handling technique. As depicted in Fig. 15b. it is obvious that the 64 load voltage buses are within the specified limits values of the load buses, and no bus experienced an overvoltage.

**Table 14:** The obtained results of the proposed LSDO as well as the original SDO techniques for IEEE 118-bus.

Control variables	SDO	LSDO	Control Variables	SDO	LSDO
P$${}_{g1}$$	40.1063	64.4748	V$${}_{g31}$$	1.0368	1.0116
P$${}_{g4}$$	43.8024	34.3105	V$${}_{g32}$$	1.0306	1.0359
P$${}_{g6}$$	55.3404	30.5358	V$${}_{g34}$$	1.0329	1.0020
P$${}_{g8}$$	55.5974	30.1350	V$${}_{g36}$$	1.0317	0.9965
P$${}_{g10}$$	192.3171	307.7051	V$${}_{g40}$$	0.9938	1.0065
P$${}_{g12}$$	73.4690	63.1787	V$${}_{g42}$$	0.9769	1.0477
P$${}_{g15}$$	30.4367	69.2999	V$${}_{g46}$$	1.0100	1.0017
P$${}_{g18}$$	30.9970	37.4440	V$${}_{g49}$$	1.0069	1.0181
P$${}_{g19}$$	44.1664	30.1235	V$${}_{g54}$$	1.0370	0.9989
P$${}_{g24}$$	57.6019	31.1649	V$${}_{g55}$$	1.0311	0.9947
P$${}_{g25}$$	119.2457	136.1038	V$${}_{g56}$$	1.0320	0.9967
P$${}_{g26}$$	151.3569	152.9985	V$${}_{g59}$$	1.0295	1.0074
P$${}_{g27}$$	56.0971	32.4025	V$${}_{g61}$$	1.0340	1.0008
P$${}_{g31}$$	32.1709	32.1071	V$${}_{g62}$$	1.0277	0.9988
P$${}_{g32}$$	51.6170	30.0514	V$${}_{g65}$$	1.0306	1.0398
P$${}_{g34}$$	32.0154	33.4394	V$${}_{g66}$$	1.0117	1.0184
P$${}_{g36}$$	31.4379	30.0830	V$${}_{g69}$$	1.0401	1.0372
P$${}_{g44}$$	32.3694	31.9403	V$${}_{g70}$$	1.0231	1.0133
P$${}_{g42}$$	30.6368	32.5210	V$${}_{g72}$$	1.0276	1.0123
P$${}_{g46}$$	36.1461	35.9978	V$${}_{g73}$$	1.0163	0.9956
P$${}_{g49}$$	112.0778	160.9314	V$${}_{g74}$$	1.0059	1.0019
P$${}_{g54}$$	62.1124	47.7883	V$${}_{g76}$$	0.9781	0.9941
P$${}_{g55}$$	54.0745	30.3659	V$${}_{g77}$$	1.0033	1.0202
P$${}_{g56}$$	44.3759	44.7771	V$${}_{g80}$$	1.0172	1.0369
P$${}_{g59}$$	145.3439	88.6009	V$${}_{g85}$$	1.0039	0.9969
P$${}_{g61}$$	117.0340	126.8220	V$${}_{g87}$$	1.0626	0.9837
P$${}_{g62}$$	45.7334	39.6513	V$${}_{g89}$$	1.0135	0.9986
P$${}_{g65}$$	284.0753	263.3131	V$${}_{g90}$$	1.0207	1.0236
P$${}_{g66}$$	172.3229	278.0789	V$${}_{g91}$$	1.0041	1.0544
P$${}_{g70}$$	50.4983	30.0481	V$${}_{g92}$$	1.0037	1.0095
P$${}_{g72}$$	32.3044	63.0797	V$${}_{g99}$$	1.0198	1.0579
P$${}_{g73}$$	49.1009	53.9478	V$${}_{g100}$$	1.0120	1.0290
P$${}_{g74}$$	55.6390	35.4073	V$${}_{g103}$$	1.0194	1.0151
P$${}_{g76}$$	59.7134	52.1510	V$${}_{g104}$$	1.0150	1.0015
P$${}_{g77}$$	30.0000	32.1093	V$${}_{g105}$$	1.0214	1.0018
P$${}_{g80}$$	282.5561	289.0136	V$${}_{g107}$$	1.0604	1.0147
P$${}_{g85}$$	30.3429	30.3009	V$${}_{g110}$$	1.0154	1.0027
P$${}_{g87}$$	31.2028	31.2072	V$${}_{g111}$$	1.0450	1.0281
P$${}_{g89}$$	370.5745	390.9766	V$${}_{g112}$$	0.9919	0.9919
P$${}_{g90}$$	63.0840	30.7519	V$${}_{g113}$$	1.0317	1.0461
P$${}_{g91}$$	40.2548	31.5360	V$${}_{g113}$$	1.0268	1.0272
P$${}_{g92}$$	50.2085	41.3658	Q$${}_{c5}$$	8.4799	9.6180
P$${}_{g99}$$	31.6909	32.3164	Q$${}_{c34}$$	12.7735	1.8728
P$${}_{g100}$$	200.5469	159.9352	Q$${}_{c37}$$	6.3689	15.4410
P$${}_{g103}$$	48.9003	47.5207	Q$${}_{c44}$$	8.4943	16.1184
P$${}_{g104}$$	30.0499	30.8410	Q$${}_{c45}$$	21.2064	17.7603
P$${}_{g105}$$	30.1693	31.3924	Q$${}_{c46}$$	11.1423	12.8668
P$${}_{g107}$$	30.1765	30.2941	Q$${}_{c48}$$	13.3364	16.5950
P$${}_{g110}$$	37.8482	30.8276	Q$${}_{c74}$$	14.0006	15.7325
P$${}_{g111}$$	76.6624	41.3898	Q$${}_{c79}$$	15.0967	12.5671
P$${}_{g112}$$	30.3786	31.8328	Q$${}_{c82}$$	8.1848	11.6904
P$${}_{g113}$$	56.1582	30.0014	Q$${}_{c83}$$	13.9846	12.7562
P$${}_{g116}$$	32.8097	30.4287	Q$${}_{c105}$$	16.3278	17.0788
V$${}_{g1}$$	1.0028	0.9925	Q$${}_{c107}$$	6.1172	1.8649
V$${}_{g4}$$	1.0138	1.0211	Q$${}_{c110}$$	15.8669	1.2406
V$${}_{g6}$$	1.0173	1.0052	T$${}_{8}$$	0.9652	0.9416
V$${}_{g8}$$	1.0256	0.9878	T$${}_{32}$$	1.0156	0.9997
V$${}_{g10}$$	1.0286	1.0168	T$${}_{36}$$	0.9894	0.9783
V$${}_{g12}$$	1.0199	0.9958	T$${}_{51}$$	0.9879	1.0174
V$${}_{g\ 15}$$	1.0041	1.0070	T$${}_{93}$$	0.9765	1.0166
V$${}_{g18}$$	1.0023	0.9997	T$${}_{95}$$	0.9761	1.0029
V$${}_{g19}$$	0.9993	0.9982	T$${}_{102}$$	1.0115	1.0237
V$${}_{g24}$$	1.0020	1.0165	T$${}_{107}$$	1.0039	1.0447
V$${}_{g25}$$	1.0438	1.0252	T$${}_{127}$$	0.9828	1.0427
V$${}_{g26}$$	1.0701	1.0078	Fuel Cost ($/h)	139923.69	**137105.99**
V$${}_{g27}$$	1.0407	1.0540	P$${}_{g69}$$	319.5107	374.0803

The best values obtained are in bold.

#### Statistical results

Table 15 summarizes the statistical comparison of 30 independent runs between SDO and its improved variant corresponding to their min, mean, max, and standard deviation (SD) of fitness values. As mentioned before, the optimal objective function value achieved in each cases by LSDO optimizer outperforms the SDO solutions. Additionally, the mean, min, and SD are as well better in almost cases. Additionally, the statistical summary presented in Table 16 comprises the min, mean, max, and SD objective values obtained from 30 independent runs. This summary clearly demonstrates that the LSDO algorithm surpasses all other re-implemented algorithms in terms of performance. Remarkably, the worst fitness values achieved by LSDO are better than the best fitness values attained by the competing algorithms (i.e. ABC and DE in all cases, AEO in cases 5 and 6, PSO in all cases except case 10). This indicates that the LSDO algorithm consistently provides superior optimization outcomes across the considered scenarios, showcasing its effectiveness and robustness.

**Table 15:** Statistical results of the proposed LSDO and original SDO algorithms for case studies without RESs.

Systems	Cases		Min	Mean	Max	SD
IEEE 30	Case 1	SDO	800.4223	800.4461	**800.4776**	1.4516E−2
		LSDO	**800.42**	**800.4404**	800.4828	**1.4232E−2**
	Case 2	SDO	0.20484	**0.20486**	0.20492	**2.3422E−5**
		LSDO	**0.20483**	0.20487	**0.20491**	2.535E−5
	Case 3	SDO	0.092494	0.095292	0.10039	2.6015E−3
		LSDO	**0.091521**	**0.093677**	**0.10008**	**2.2197E−3**
	Case 4	SDO	3.0908	3.0983	3.1267	1.0211E−2
		LSDO	**3.0902**	**3.0932**	**3.1024**	**2.6589E−3**
IEEE 57	Case 6	SDO	41668.7587	41669.5291	41676.463	2.4363
		LSDO	**41667.719**	**41667.807**	**41667.915**	**1.001E−1**
	Case 7	SDO	0.63354	0.64428	0.66422	1.5011E−2
		LSDO	**0.62165**	**0.63646**	**0.64016**	**7.5964E−3**
	Case 8	SDO	10.4552	10.5735	11.0571	1.8523E−1
		LSDO	**10.2332**	**10.2551**	**10.6719**	**9.8104E−2**
IEEE 118	Case 12	SDO	139923.69	140792.50	144232.38	1588.4573
		LSDO	**137105.99**	**137354.50**	**138858.69**	**576.4613**

Significant values are in bold.

After a meticulous examination of the results obtained from evaluating different aspects and objectives across 23 benchmark functions and three distinct test networks, it has been established that the LSDO algorithm excels in effectively addressing the OPF problems compared to other alternative methods. Worth noting is that the considered cases represent diverse scenarios and conditions in power system, encompassing a wide range of complexities. Despite the varying characteristics of cases, the LSDO consistently exhibited superior performance in terms of convergence and attaining optimal solutions. The comparison was based on various metrics, including fitness values, convergence rates, and constraint satisfaction, all of which further support the robustness and effectiveness of the LSDO algorithm in solving the optimization challenges in the power systems domain.

**Table 16:** Statistical results of the proposed LSDO and its competitors for case studies with RESs.

Systems	Cases		LSDO	SDO	ABC	AEO	DE	PSO
IEEE 30	Case 5	Min	**781.0465**	781.0633	781.5679	781.5613	781.8429	781.8984
		Mean	**781.0777**	781.1211	781.6922	781.7967	781.9374	785.7463
		Max	**781.1041**	781.3601	781.9680	782.8320	782.0656	813.5669
		SD	**1.8922e−02**	7.9737e−02	9.9345e−02	2.5226e−01	5.4209e−02	5.5157e+00
		p_value	N/A	7.2253e−02	2.7792e−11	2.7792e−11	2.7792e−11	2.7792e−11
	Case 6	Min	**809.5379**	809.5406	810.2063	810.3869	810.3891	810.6643
		Mean	809.5686	**809.5534**	810.2226	810.4587	810.4189	812.5426
		Max	**809.6433**	809.6613	810.2596	810.5989	810.4526	828.2575
		SD	2.4758e−02	2.9860e−02	**1.2469e−02**	6.0906e−02	1.7633e−02	3.1834e+00
		p_value	N/A	2.1627e−05	2.8538e−11	2.8538e−11	2.8538e−11	2.8538e−11
IEEE 57	Case 10	Min	**26491.8072**	26529.4584	30539.0423	26496.6212	27458.7947	26565.5122
		Mean	**26509.1985**	26544.8073	35120.4814	29735.3255	29253.3064	34947.4702
		Max	**26571.5093**	26577.9994	40518.7080	41637.2155	32302.3813	56398.4406
		SD	2.0740e+01	**1.6977e+01**	2.5649e+03	3.4953e+03	1.1643e+03	7.6620e+03
		p_value	N/A	4.0707e−08	2.2204e−11	1.9203e−08	2.2204e−11	2.4579e−11
	Case 11	Min	**26490.4174**	26560.9184	28755.3530	26500.9984	27247.6331	26695.5204
		Mean	**26513.1831**	26561.6940	35555.4767	29647.0590	29235.2903	33617.4824
		Max	**26547.1563**	26563.0338	51597.0759	45841.5302	31924.7822	54730.8211
		SD	2.4701e+01	**1.0368e+00**	4.9651e+03	4.5404e+03	1.1092e+03	6.8201e+03
		p_value	N/A	9.9140e−12	2.4446e−11	6.0580e−09	2.4446e−11	2.4446e−11

Significant values are in bold.

## Conclusion

This paper presents an ameliorate SDO algorithm for solving one of the power system issues considering renewable energy powers. In order to confirm the effectiveness of this algorithm, a set of test functions have been employed to benchmark the performance of the LSDO approach from different perspectives; then, three power system models and different case studies were investigated. The improved algorithm LSDO-based SF constraint handling method has been accomplished successfully and proves the utility of the SF strategy in dealing with the systems’ constraints. Accordingly, the obtained statistical results confirm the efficiency and capability of the LSDO in getting the best solutions, as it outstripped the standard version SDO and the well-known approaches TSA, GWO, SNS, ABC, DE, AEO, and PSO. Along these lines, this proposed LSDO algorithm has the ability to handle the various drawbacks of its basic algorithm, in terms of balancing between the exploration and exploitation processes. Furthermore, the LSDO convergence feature appears to have improved and shows a reasonable convergence speed to the fitness value than its initial version. In addition, the comparative study of LSDO, SDO and competitor algorithms affirms the potential of LSDO in finding accurate solutions notably for large-scale power systems and solving constrained non-linear complex real-world problems. In accordance with these remarkable outcomes, the authors recommend an LSDO-based SF strategy to handle the OPF issue for a realistic and higher dimension as considered in this present research.

## Data Availability

The datasets generated during and/or analyzed during the current study are available from the corresponding author on reasonable request.
